# Fast near-whole–brain imaging in adult Drosophila during responses to stimuli and behavior

**DOI:** 10.1371/journal.pbio.2006732

**Published:** 2019-02-15

**Authors:** Sophie Aimon, Takeo Katsuki, Tongqiu Jia, Logan Grosenick, Michael Broxton, Karl Deisseroth, Terrence J. Sejnowski, Ralph J. Greenspan

**Affiliations:** 1 Kavli Institute for Brain and Mind, UCSD, La Jolla, California, United States of America; 2 Computational Neurobiology Laboratory, Salk Institute for Biological Studies, La Jolla, California, United States of America; 3 Neurobiology Section, University of California, San Diego, La Jolla, California, United States of America; 4 Departments of Computer Science and Bioengineering, Stanford University, Stanford, California, United States of America; 5 Departments of Bioengineering and Psychiatry, Stanford University, Stanford, California, United States of America; 6 Howard Hughes Medical Institute, Stanford University, Stanford, Stanford, California, United States of America; 7 Division of Biological Sciences, University of California San Diego, La Jolla, California, United States of America; 8 Department of Cognitive Science, University of California, San Diego, La Jolla, California, United States of America; Stony Brook University, United States of America

## Abstract

Whole-brain recordings give us a global perspective of the brain in action. In this study, we describe a method using light field microscopy to record near-whole brain calcium and voltage activity at high speed in behaving adult flies. We first obtained global activity maps for various stimuli and behaviors. Notably, we found that brain activity increased on a global scale when the fly walked but not when it groomed. This global increase with walking was particularly strong in dopamine neurons. Second, we extracted maps of spatially distinct sources of activity as well as their time series using principal component analysis and independent component analysis. The characteristic shapes in the maps matched the anatomy of subneuropil regions and, in some cases, a specific neuron type. Brain structures that responded to light and odor were consistent with previous reports, confirming the new technique’s validity. We also observed previously uncharacterized behavior-related activity as well as patterns of spontaneous voltage activity.

## Author summary

Whole-brain recordings give us a global perspective of the brain in action. This is already possible in humans, for which functional magnetic resonance imaging (fMRI) has opened a new chapter in the study of brain activity underlying behavior, but this technique has low spatial and temporal resolution. In animals, techniques for imaging a whole brain so far have allowed us to record activity at much higher spatial resolution, but these are still orders-of-magnitude slower than neuronal electrical activity. Here, we have developed a technique for ultra-fast imaging of whole-brain activity in fruit flies while they are behaving (walking, resting, or grooming) and when they perceive various stimuli. We find that there is a global increase in activity when the fly walks compared to when it rests, while only a small local increase is observed when the fly grooms compared to when it rests. We have also used computational techniques to extract activity from small brain regions or from specific neuron types and identified regions involved in turning left or right as well as regions with ongoing activity in the absence of stimuli or behavior.

## Introduction

Measuring activity simultaneously in the whole brain is critical to understanding how different brain regions interact to process and control sensory inputs, internal states, and behavior. Whole-brain recordings not only reveal which regions are involved in which functions and with what network dynamics but also help to interpret the effects of a targeted intervention (e.g., a lesion or a local alteration with optogenetics) on the whole network and give context to local electrophysiology recordings. Furthermore, they are necessary to characterize global changes affecting the brain on a large scale (such as different behavioral states) and detect patterns of activity involving distant regions.

This is already possible in humans, for which functional magnetic resonance imaging (fMRI) has opened a new chapter in the study of brain activity underlying behavior, but this technique has low spatial and temporal resolution. In animals, techniques for imaging a whole brain so far have allowed us to record activity at much higher resolutions but are still orders-of-magnitude slower than neuronal electrical activity. For example, recent reports of scanning-based whole-brain imaging in zebrafish and Drosophila larvae had a frame rate of 12 Hz [1] and 5 Hz [2], respectively. By contrast, light field microscopy [3–9] makes it possible to image large volumes of scattering brain tissue at more than 100 Hz. In this study, we leverage this technique to record large-scale activity in the brain of behaving adult fruit flies. We present a method to optically access the fly’s brain while enabling it to retain the ability to walk or groom. We show that the near-whole brain can be imaged with a 20x objective at a frame rate up to 200 Hz and fluorescence recorded from pan-neuronally expressed calcium (GCaMP6 [10]) or voltage (ArcLight [11]) probes. We present rich data sets of near-whole brain activity and behavior as well as two analysis methods. First, we map activity for specific stimuli and behaviors with short time-scales; for example, we compared activity when the fly rested, walked, and groomed. Second, we apply a computational method (principal component analysis [PCA], followed by independent component analysis [ICA]) to extract components representing spatially distinct sources of activity [6,12,13]. We show that these sources correspond to subneuropil areas or processes from small populations of neurons that are anatomically well characterized, and we compare their responses to flashes of light or odor puffs with those in literature reports of experiments done on restricted regions. Additionally, by using this method, we discovered neuronal projections whose activity correlated with turning as well as previously unreported patterns of spontaneous voltage activity.

## Results

### Imaging the near-whole brain of behaving adult Drosophila

**Fig 1:**
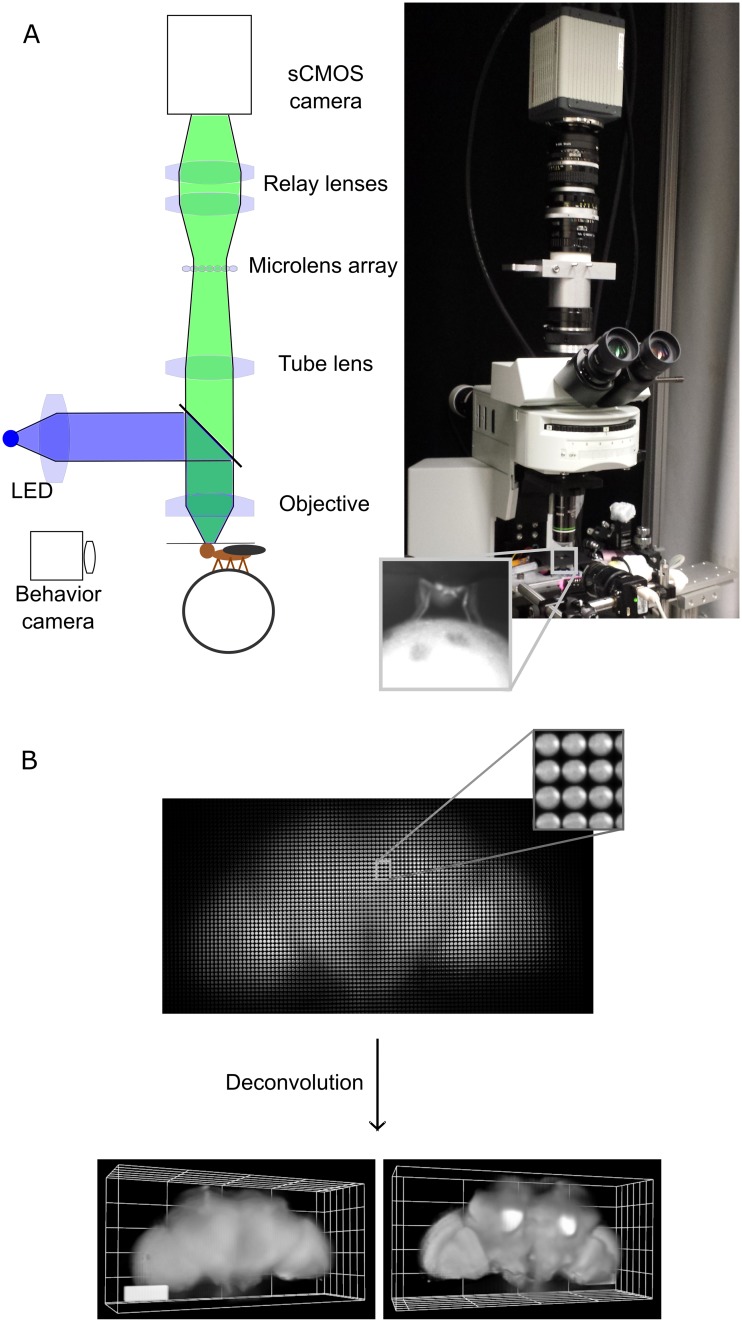
Imaging the brain of adult behaving flies using light field microscopy. A) Experimental setup. The fly is head fixed, and its tarsi are touching a ball. The light from the brain’s fluorescence goes through the objective, the microscope tube lens, a microlens array, and relay lenses onto the sensor of a high-speed sCMOS camera. Another camera in front of the fly records its behavior. B) Example of a light field deconvolution (fly’s genotype: *nsyb-Gal4*, *UAS-ArcLight*). Top: 2D light field image acquired in 5 ms—one camera acquisition period—with a 20x NA 1.0 objective. Bottom: anterior and posterior views (slightly tilted sideways) of the computationally reconstructed volume. 3D bar is 90 x 30 x 30 microns. See also S1–S3 Figs. NA, numerical aperture; *nysb-Gal4*, nSynaptobrevin-Gal4; sCMOS, scientific complementary metal-oxide-semiconductor.

We first fixed a fly’s head by rotating it 45 degrees or more around the transversal axis to decrease the depth of the volume imaged and to improve access to the brain. We then exposed the brain while keeping the eyes, antennae, and legs intact and clean (see Methods section and S1 Fig). A ball was placed under the fly’s tarsi so that it could typically rest, walk, and groom. We imaged the fly brain’s fluorescence using light field microscopy. As shown in Fig 1A, we modified an upright epifluorescence microscope (equipped with a 20x 1.0 numerical aperture [NA] or a 40x 0.8 NA objective) by adding a microlens array at the image plane of the objective and placing the camera sensor at the image plane of the microlens array through relay lenses. We recorded light field images continuously with a high-speed scientific complementary metal-oxide-semiconductor (sCMOS) camera up to 100 Hz for GCaMP6 and 200 Hz for ArcLight (using the middle of the camera sensor).

We then reconstructed the volumes—typically 600 x 300 x 200 μm^3^ to encompass the whole brain (Fig 1B)—using the volumetric deconvolution method for light field microscopy described in [3]. Note that unlike other microscopy techniques that are based on scanning (e.g., two-photon, confocal, or light-sheet microscopy), excitation light illuminates the entire brain continuously, and all the photons emitted in the numerical aperture of the objective are used to reconstruct the image (minus an approximately 40% loss through the objective, tube lens, microlens array, and relay lenses). This maximizes the number of photons collected (and thus information about brain activity) per units of volume and time.

We used 2-μm fluorescent beads embedded in a gel to measure the point spread function and found that it widens with distance from the focal plane, varying from 3.5 to 12 μm laterally and from 6 to 35 μm axially for 20x 1.0 NA objective and varying from 2 to 7 μm laterally and from 4 to 22 μm axially for 40x 0.8 NA objective (S2 Fig and theoretical expression in [3]). As shown in S3 Fig (presenting ArcLight’s baseline fluorescence) and below, this resolution was sufficient to recognize neuropil structures and extract activity from subneuropil compartments.

### Global activity during response to stimuli and behavior

**Fig 2:**
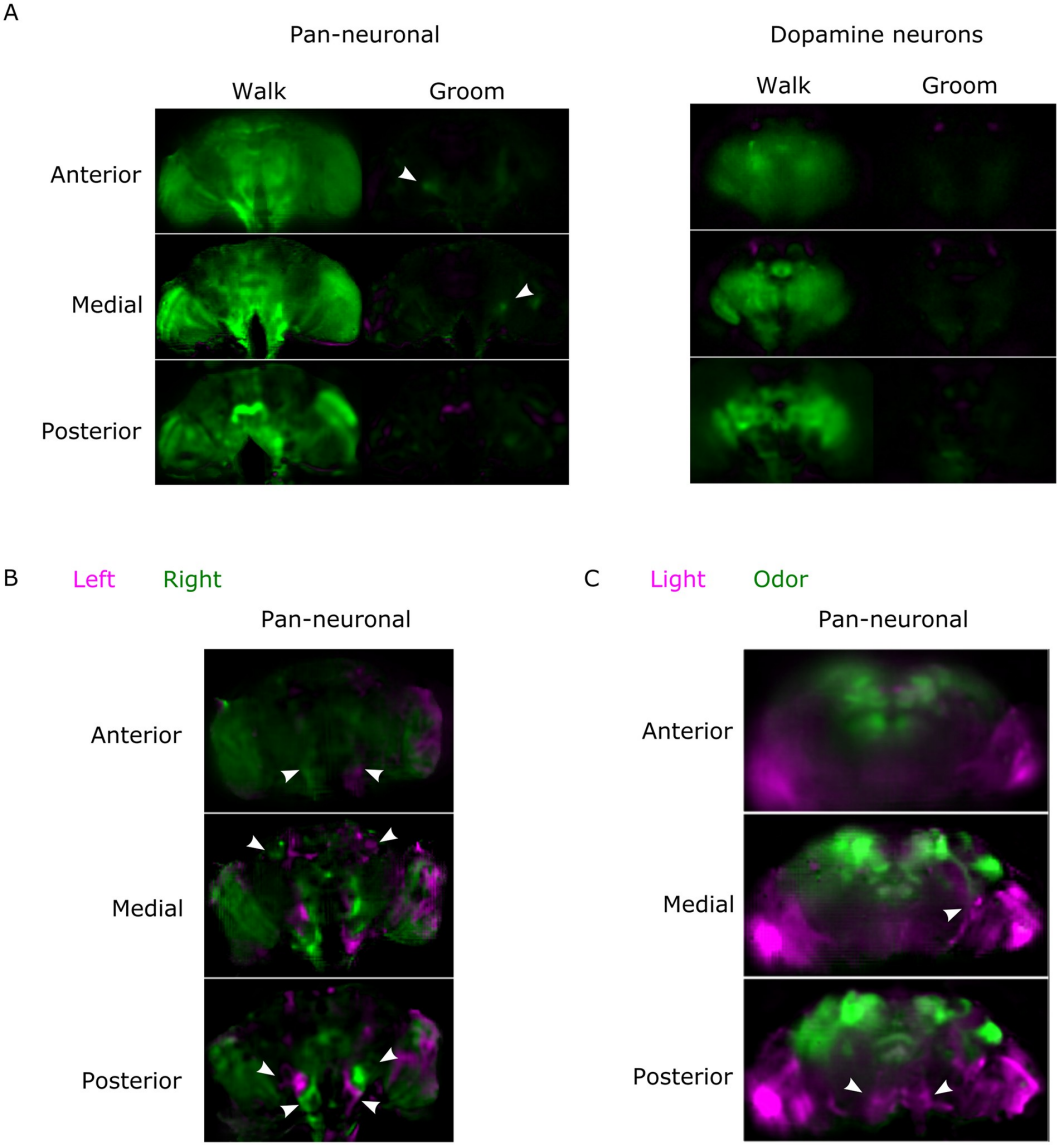
Near-whole brain activity maps for various conditions. A) Comparison of fluorescence intensity when the fly rests and when it is active (either walking or grooming). The pixel value is green if the fluorescence is higher during the behavior and magenta if the fluorescence is higher during rest. Arrows point to regions (in the antennal mechanosensory and motor center) corresponding to the higher average change between groom and rest among flies (see also S9 Fig). The pan-neuronal driver was *nsyb-Gal4* and the dopamine driver was *TH-Gal4*. Both flies expressed GCaMP6f. Note that there is an angle (39 degrees for the pan-neuronal experiment and 20 degrees for the dopamine experiment) between the z-axis and the anterior—posterior axis. B) Comparison of fluorescence intensity when the fly turns left or right. The arrows point to regions (in the posterior slope, gnathal ganglia, and superior lateral protocerebrum or anterior tubercule) symmetrical and reproducible from fly to fly. The fly’s genotype was *nsyb-Gal4* and *UAS-GCaMP6f*. Note that there is a 40 degree angle between the z-axis and the anterior—posterior axis. The pixel value is green if the fluorescence is higher during turning left, magenta if the fluorescence is higher during turning right. C) Comparison of fluorescence increase during the response to stimuli for odor (magenta) and light (green). The arrows point to the posterior slope (bottom panel) and the optical glomeruli (middle panel). The fly’s genotype was *Cha-Gal4*, *GMR57C10-Gal4*, and *UAS-GCaMP6f*. Note that the brain is tilted 19 degrees along the lateral axis. See also S4–S9 Figs. Data can be found on CNCRS.org (http://dx.doi.org/10.6080/K01J97ZN). CNCRS, Collaborative Research in Computational Neuroscience; *nsyb-Gal4*, nSynaptobrevin-Gal4; *TH-Gal4*, tyrosine hydroxylase-Gal4; *Cha-Gal4*, choline acetyltransferase-Gal4.

S1 and S2 Movies present maximum z projections of near-whole brain activity (after preprocessing as described in S4 Fig and the Methods section) when stimuli were presented to the fly. Fig 2C and S5C Fig show maps of the response to stimuli. We found strong increases in activity at the onset of puffs of odor and flashes of UV light in specific parts of the brain (see S5–S7 and S9 Figs), in accordance with previous reports in the literature: the strongest responses to light involved the optic lobes, optical glomeruli, and posterior slope, and the responses to odor involved the antennal lobe and most of the dorsal neuropils including the lateral horn, superior neuropils, and the mushroom body (S5C, S6, S7 and S9 Figs). The global map of the response to stimuli was similar for calcium (GCaMP6) and voltage (ArcLight) activity (S5C and S9 Figs).

We also examined near-whole brain activity in the absence of external stimuli (i.e., spontaneous behavior), which consisted of walking, grooming, and resting (see S3–S7 Movies). Most strikingly, the brain was more active on a global scale when the fly walked than when it rested or groomed (see S3 Movie, Fig 2A, and S5A, S8, and S9 Figs), and global activity (average ΔF/F) was correlated with walking (R^2^ = 0.37 +/− 0.19, average +/− STD, *N* = 6). To verify that this response was linked to walking rather than the optic flow from the ball, we repeated the experiment with a visually impaired *norpA* mutant fly and again found a global increase during walking in comparison with rest (see S4 Movie and S5A Fig). In contrast, we found only local activation in the region of the saddle, wedge, and antennal mechanosensory and motor center (in four out of five flies) during grooming. To investigate whether the global increase during walking was coming exclusively from neurons expressing one type of neurotransmitter or neuromodulator, we performed the same experiments with more restricted lines. We also found a global increase when GCaMP6f was expressed in cholinergic neurons, which form the majority of excitatory neurons in the fly brain (R^2^ = 0.49 for the correlation of average ΔF/F with walk) (S5 Movie and S5A Fig). When GCaMP6f was expressed in dopamine neurons only (with *TH-Gal4*: S6 Movie, Fig 2A, and S5A Fig), we observed a strong large-scale increase of activity tightly locked with walking. We also found surprisingly little activity during resting or grooming, apart from the mushroom body area. This increase was observed in flies expressing GCaMP6f in both dopamine and serotonin neurons (*TH-Gal4* and *DDC-Gal4*), but in that case, we also observed more background activity unrelated to behavior (S7 Movie and S5A Fig).

We investigated the difference in activity when the fly turned left compared to right (Fig 2B and S5B Fig). Although there was a strong variability from fly to fly that will need to be characterized in future studies, we observed antisymmetric patterns in the ventral areas and in the lateral superior protocerebrum (as indicated by the arrows) in all flies.

### Source-extraction algorithm reveals functional maps at the subneuropil level

**Fig 3:**
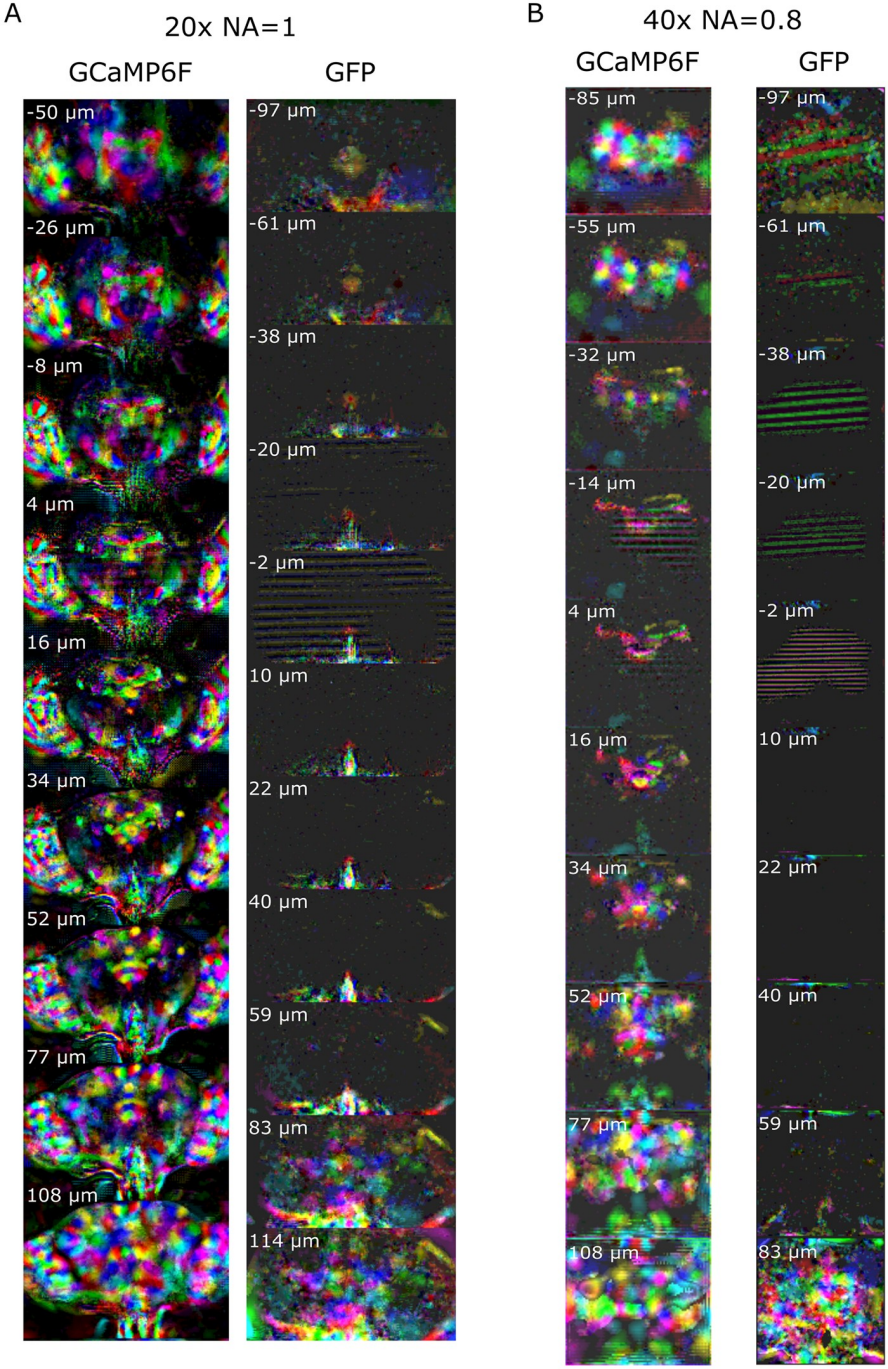
Z-stack slices of the 3D map for all the components extracted using PCA/ICA. The maps from calcium activity (GCaMP6f) are compared with the maps obtained with an activity-independent fluorophore (GFP). Different colors are assigned randomly to different components. Note that the slice depth is larger when it is farther from the middle of the brain (see Methods section). See S10 and S11 Figs for maps aligned to an anatomical template and S12 Fig for examples of individual artifactual components. GCaMP6f data can be found on CNCRS.org (http://dx.doi.org/10.6080/K01J97ZN). CNCRS, Collaborative Research in Computational Neuroscience; GFP, green fluorescent protein; ICA, independent component analysis; PCA, principal component analysis.

We used a combination of statistical methods to extract maps and time series of spatially distinct sources of activity (see Methods section for details). Briefly, we first applied PCA (using singular value decomposition) to find maps of correlated activity and to reduce dimensionality. We then applied ICA to unmix the PCA maps and find sparse functional regions (see analysis pipeline in S4 Fig). Fig 3 shows z-stacks containing all components (z-score > 3) from pan-neuronal GCaMP6 recordings (see also additional flies in S10 and S11 Figs). We also performed the same analysis with flies that pan-neuronally expressed activity-independent green fluorescent protein (GFP) to help identify artifact-related components—movement, aliasing, or noise, as shown in S12 Fig.

**Fig 4:**
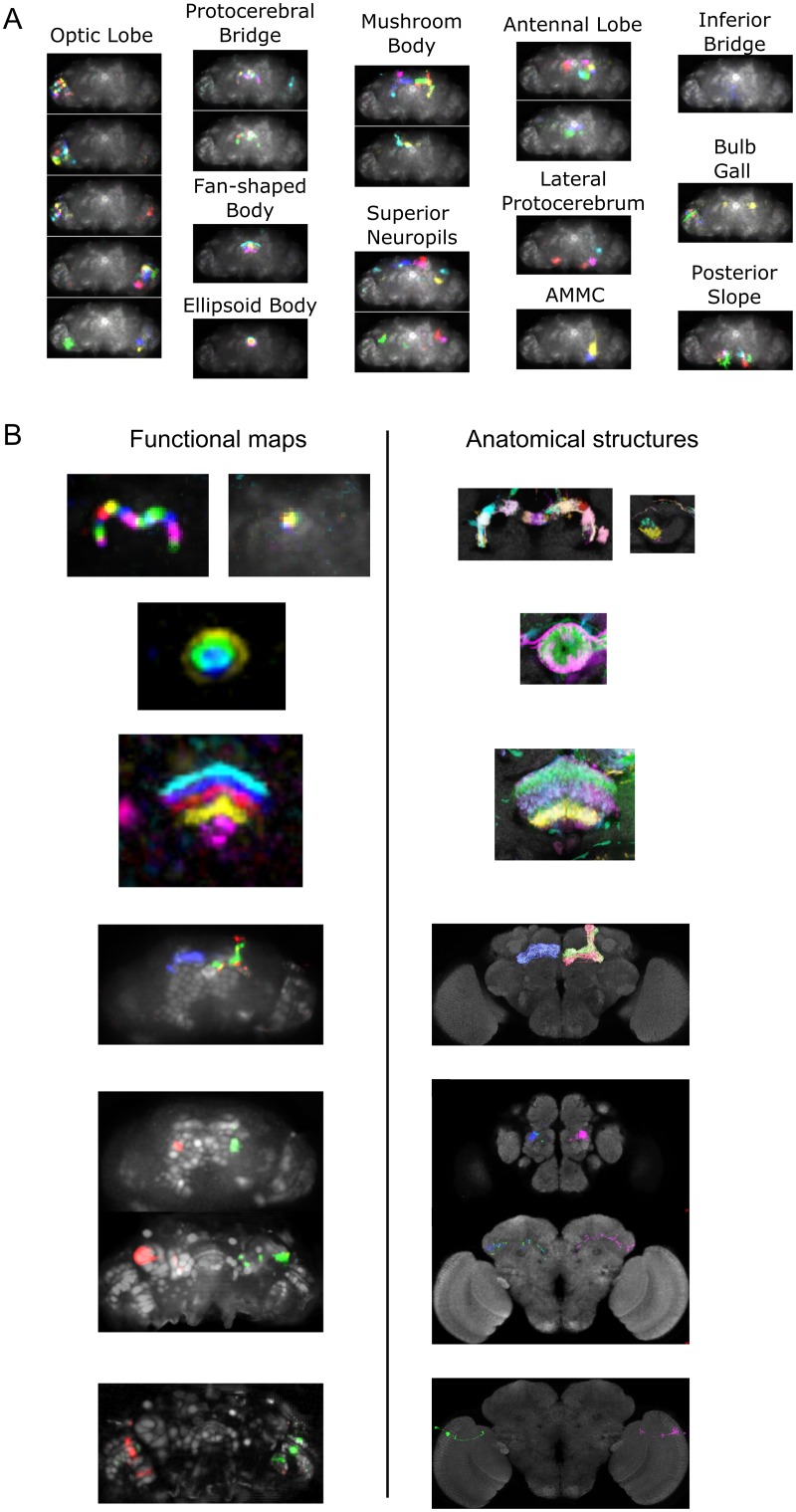
Functional components match anatomical structures. A) Components automatically sorted by region and projected along the z-axis. Note that the sorting (the component’s maps are averaged in anatomical regions of interest) could be inaccurate in the case of maps containing small parts of large regions and noise in very small regions (i.e., the bulb or gall). Data can be found on CNCRS.org (http://dx.doi.org/10.6080/K01J97ZN). B) Comparison between functional and anatomical maps. Left: functional maps from a pan-neuronal (*GMR57C10-Gal4* and *Cha-Gal4*) GCaMP6f experiment. Right: corresponding anatomical structures. Maps were constructed using the Virtual Fly Brain database. Three bottom images show neurons from major neuron types (three different Kenyon cell types, antennal lobe projection neuron, and transmedullar neurons) matching the functional maps. The brain for the functional data is tilted 19 degrees along the lateral axis compared to the template presented on the right. Note that functional maps in the fan-shaped body are the same scale as functional maps obtained with microscopy techniques with higher resolution [17]. See also S13–S17 Figs for consideration regarding the associated time series. *Cha-Gal4*, choline acetyltransferase-Gal4; CNCRS, Collaborative Research in Computational Neuroscience.

Even though PCA and ICA are mathematical algorithms that make minimal assumptions about the brain, most functional maps matched well with anatomical structures. We aligned the brain with an anatomical template [14] using landmarks registration and automatically sorted the components by brain region (Fig 4A). In Fig 4B, the left column presents the component’s thresholded maps, whereas the right column presents central complex structures or neuronal processes from [15], assembled using Virtual Fly Brain [16]. Several subneuropil regions are recognizable from the shape of the maps (e.g., protocerebral bridge glomeruli, ellipsoid body rings, and fan-shaped body layers).

For some components, the combination of subneuropil regions present in the map allowed to assign it to a specific major neuron type (see Fig 4B, bottom part). For example, z-scored maps with signals spanning both the alpha and the beta mushroom body lobes likely resulted from activity in alpha/beta Kenyon cell axons, maps with signals spanning both the alpha’ and the beta’ mushroom body lobes likely resulted from activity in alpha’/beta’ Kenyon cell axons, whereas maps with signal in the gamma lobe likely resulted from activity in the gamma Kenyon cell axons. Likewise, z-scored maps containing signals in one antennal lobe subneuropil region (with a glomerulus-like shape), in the calyx and in the lateral horn likely resulted from the activity of antennal lobe projection neurons. Maps spanning a medulla column and a lobular layer were likely from transmedullar neurons. Finally, the maps of the components matching the protocerebral bridge glomeruli also often contained radial parts of the ellipsoid body (e.g., Fig 4B, left column, top panel), suggesting that these components might originate from tile or wedge neurons [18,19].

### Extracted sources’ time series retrieve known physiology in response to stimuli and reveal projections involved in turning left or right during walking

The component’s time series (resulting from PCA/ICA or from region of interest [ROI] ΔF/F averages; S13 and S15–S17 Figs) were consistent with previous reports of activity from the brain structures identified in the components’ maps. Most of the components responding to the onset and/or offset of light were in the optic lobe [20] (S14 and S15 Figs). In contrast, components responding to puffs of odors were mostly in the antennal lobe, the lateral horn, and the mushroom body [21] (S14 and S16 Figs). Components likely representing the activity of antennal projection neurons were spontaneously active in the absence of odor, but some of them became more active when the odor was presented (S14C and S16A Figs), consistent with previous reports [22].

**Fig 5:**
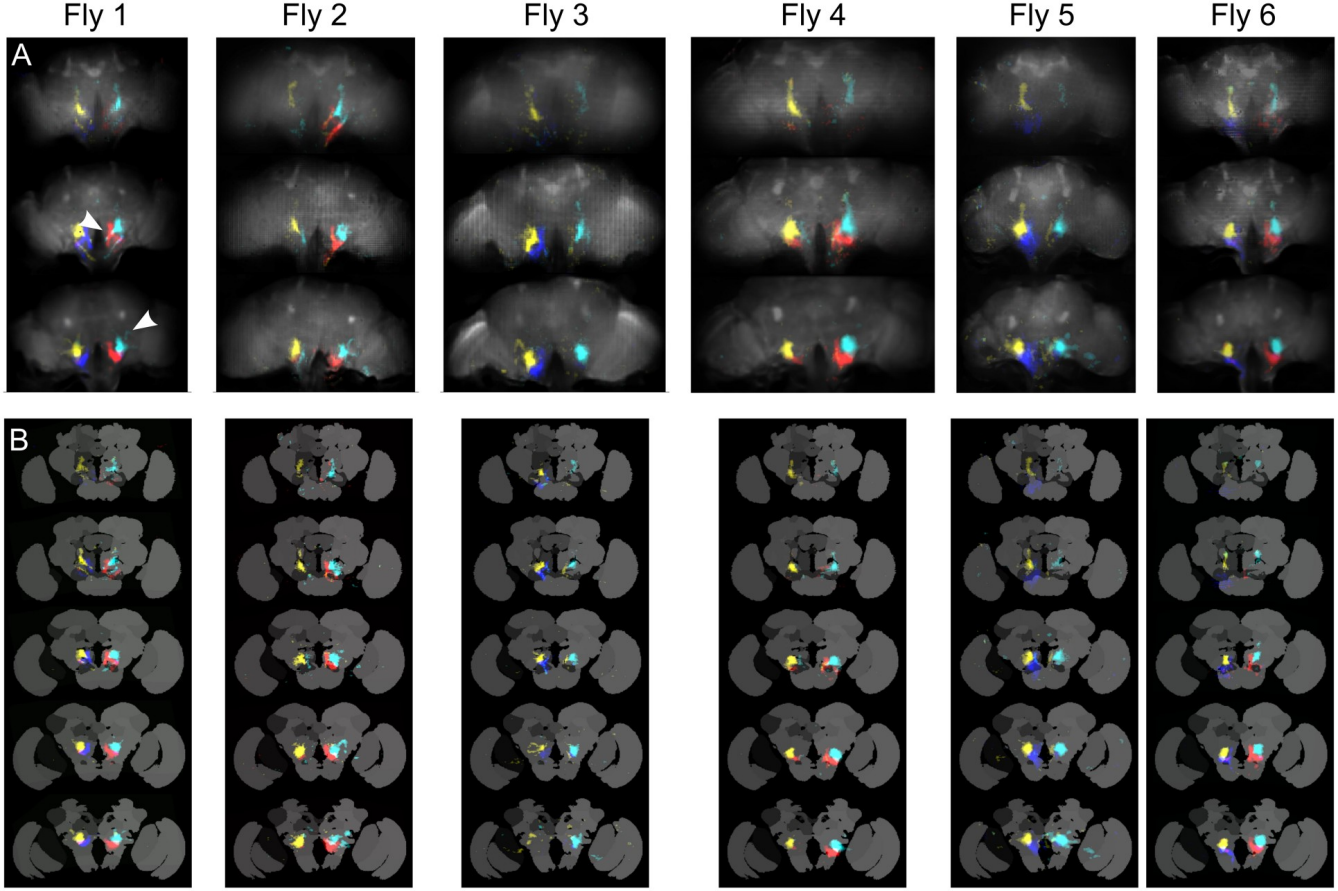
Z-stacks of components among the six most correlated to turning left (cyan and blue) or right (red and yellow). Each color corresponds to one component. Flies expressed GCaMP6 pan-neuronally. A) Anterior, medial, and posterior slices in the original orientation. The arrows point to characteristic shapes (inverted V for blue and red and fine claw-like dorsal neurites for yellow and cyan). B) The same components aligned to an anatomical template’s z-stack. See also S18 Fig. Data can be found on CNCRS.org (http://dx.doi.org/10.6080/K01J97ZN). CNCRS, Collaborative Research in Computational Neuroscience.

In addition, regions in the lateral protocerebrum, the posterior slope, the antennal mechanosensory and motor center (AMMC), the saddle, and the protocerebral bridge were strongly active when the fly walked (S17 Fig). This is consistent with previous anatomical studies; the projection from the descending neurons are most dense in the posterior slope, ventrolateral protocerebrum, and the AMMC [23]. Some of these walking-related components were strongly correlated with turning left or right (see Fig 5; Pearson’s R = 0.63 ± 0.16, mean ± SD, *N* = 12 cyan or yellow components, and Pearson’s R = 0.59 ± 0.20, mean ± SD, *N* = 8 blue or red components). Their distribution in the brain as well as strong structural characteristics (e.g., tracks forming an inverted “V” shape, fine dorsal claw-like neurites) suggest that they were generated by the same neurons across flies. Furthermore, the V-shaped components always preceded the other component correlated with turning toward the same direction—the peak of the cross-correlation function was at 68 ms for GCaMP6F (SEM = 11 ms, *N* = 6 pairs of components). As shown in S18 Fig, we found similar components when using a *Cha-Gal4* driver instead of a pan-neuronal driver (average cross-correlation peak time was 80 ms), which suggests that those neurons are cholinergic. Note that these components are mostly present in the posterior slope, as are neurons involved in turning during flight [24].

### Restricted drivers’ functional maps match single neuron’s anatomy

**Fig 6:**
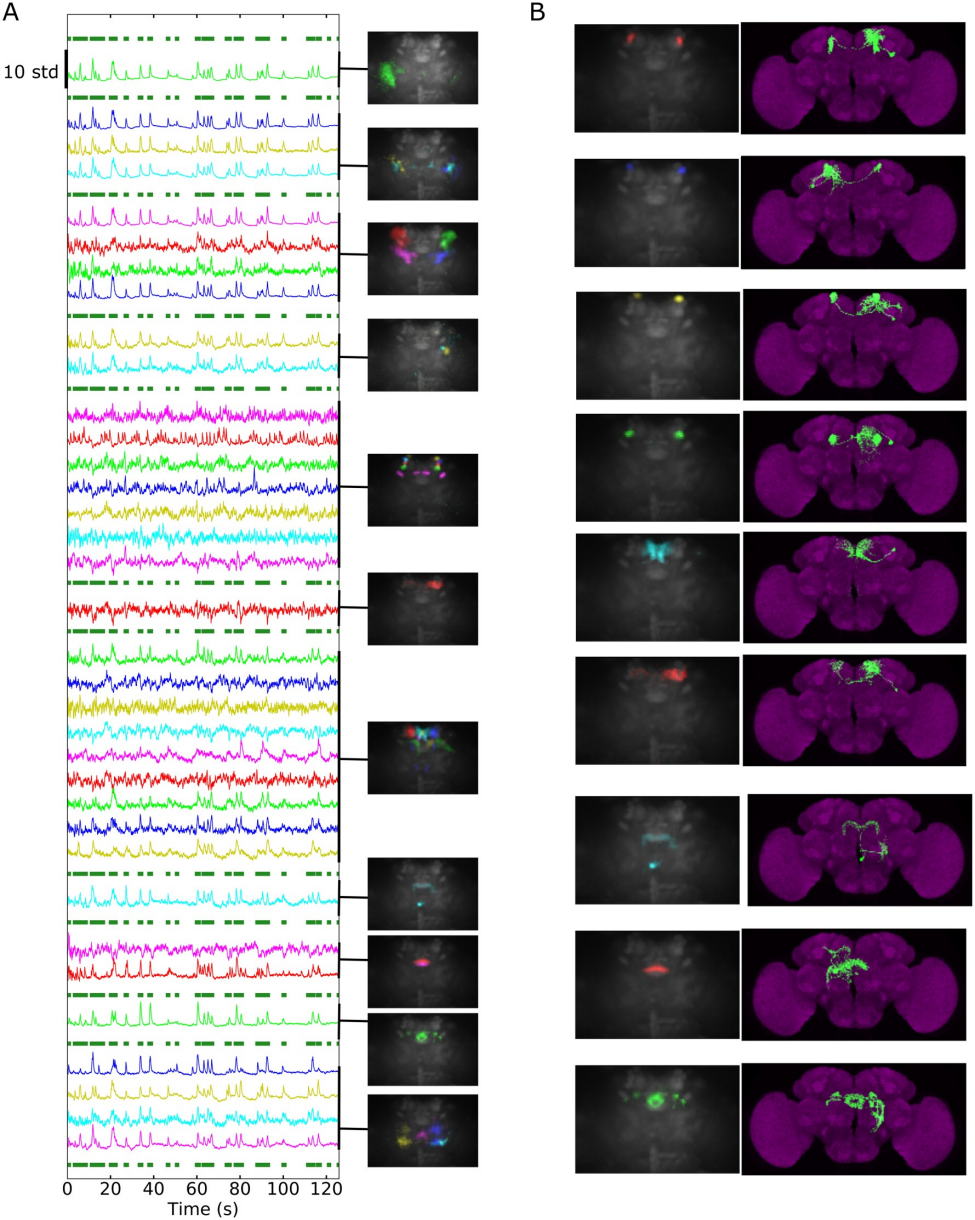
Components from flies expressing GCaMP6f in dopamine neurons (*TH-Gal4* driver). A) All activity-related components are presented and sorted by brain region, with the color of the time series (which are variance normalized) on the left, matching the color of the maps on the right (e.g., the first image corresponds to the first trace, the second image to the next three traces, and so on). Note that most components are strongly correlated with the fly walking (forest green traces interleaved with the component traces). The fly was resting or grooming the rest of the time. Data can be found on CNCRS.org (http://dx.doi.org/10.6080/K01J97ZN). B) Example of TH-positive neuron from the Virtual Fly Brain database (right) matching the components’ maps (left). Note that the brain is tilted 20 degrees along the lateral axis compared to the template presented on the right. (See also S19 Fig). CNCRS, Collaborative Research in Computational Neuroscience; *TH-Gal4*, tyrosine hydroxylase-Gal4; *Cha-Gal4*, choline acetyltransferase-Gal4.

Fig 6 and S19 Fig show components obtained when using a more restricted driver, expressing in dopamine neurons only: *TH-Gal4* (data are the same as in S6 Movie). As shown in Fig 6A, in agreement with our observation from S6 Movie, most components were tightly correlated with the fly walking (forest green traces interleaved with the components’ traces). Fig 6B reproduces some of the maps in Fig 6A along with anatomical maps of single dopaminergic neurons from the Virtual Fly Brain database [16]. Some maps had unequivocal anatomical counterparts. For example, the first three maps matched well with the anatomy of processes from dopaminergic PPL1 neurons innervating mushroom body vertical lobe compartments, each component thus corresponding to only one or two cells per hemisphere [25].

### Components from pan-neuronal voltage recordings

**Fig 7:**
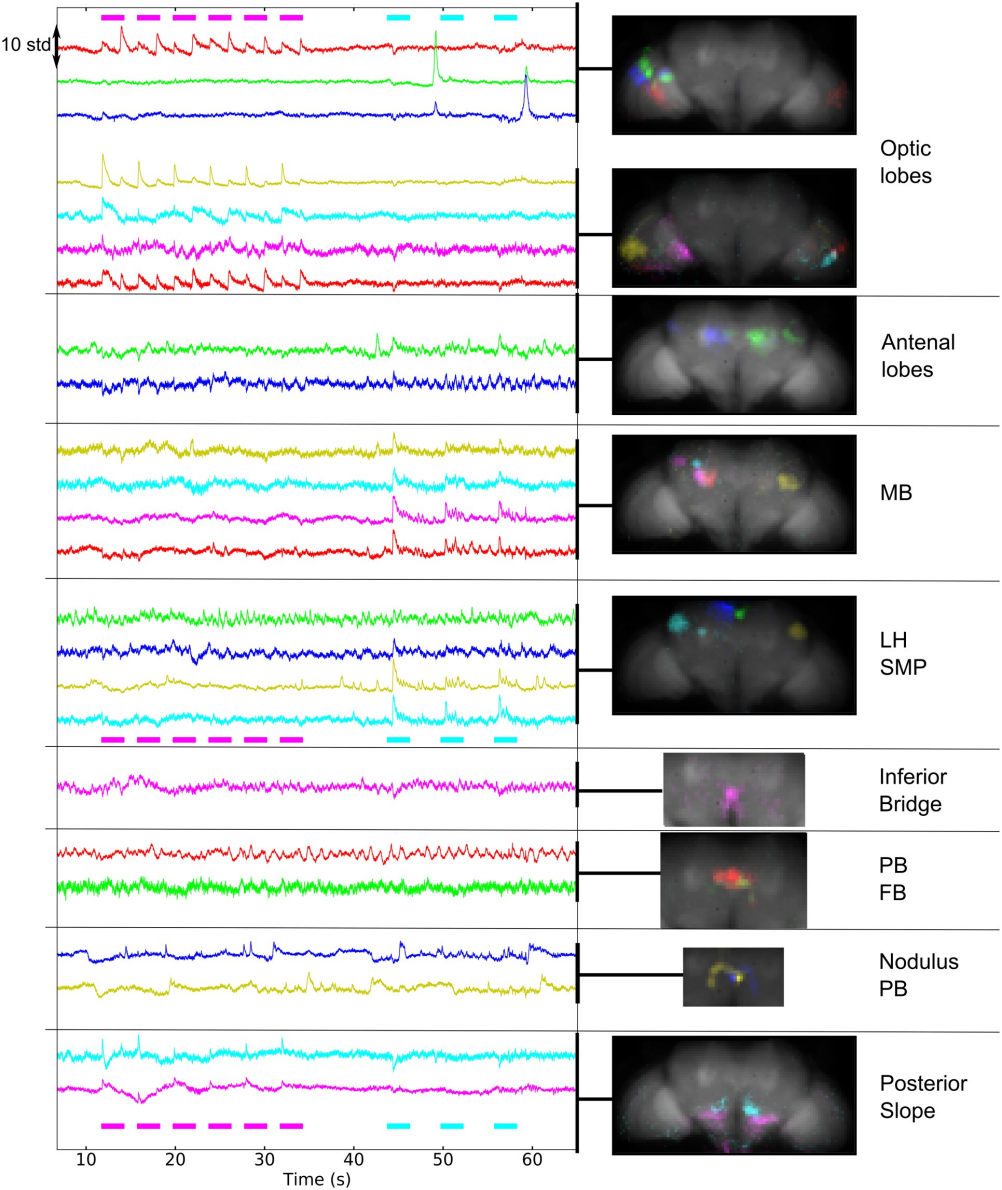
Components extracted from voltage activity. ArcLight was expressed pan-neuronally (*nsyb-Gal4*). The fly was presented with periodic flashes of UV light (violet bars) and puffs of apple cider vinegar (pink bars). The component time series are shown on the left (variance normalized), and the corresponding maps are on the right, sorted by the brain region that was majorly present in the map. Note that the coronal plane was tilted 37 degrees away from the horizontal plane for this fly. See also S20–S23 and S25 Figs. Data can be found on CNCRS.org (http://dx.doi.org/10.6080/K01J97ZN). CNCRS, Collaborative Research in Computational Neuroscience; FB, fan-shaped body; LH, lateral horn; MB, mushroom body, *nsyb-Gal4*, nSynaptobrevin-Gal4; PB, protocerebral bridge; SMP, superior medial protocerebrum.

As Fig 7 and S20 Fig demonstrate, voltage recordings with ArcLight also gave rise to maps portraying specific neuropils (and clearly distinguishable from artifacts shown in S21 Fig). As S22 Fig shows, the number of components per brain region was typically smaller than it was for GCaMP6—we extracted an average 174 (STD = 68, *N* = 12) activity-related components (i.e., not noise or movement artifacts as detailed in the Methods section) from GCaMP6 recordings and 54 (STD = 14, *N* = 6) from ArcLight recordings, probably because of the probe’s lower signal-to-noise ratio. However, ArcLight components were similar to those found with GCaMP6: in the optic lobe, some components responded to the onset and/or offset of light, with various degrees of habituation. In the posterior slope, we found peaks at the onsets of light. We also recorded large peaks of activity in the ventrolateral protocerebrum. Finally, we found components in the antennal lobe, lateral horn, and mushroom body responding to odor (S23 Fig).

**Fig 8:**
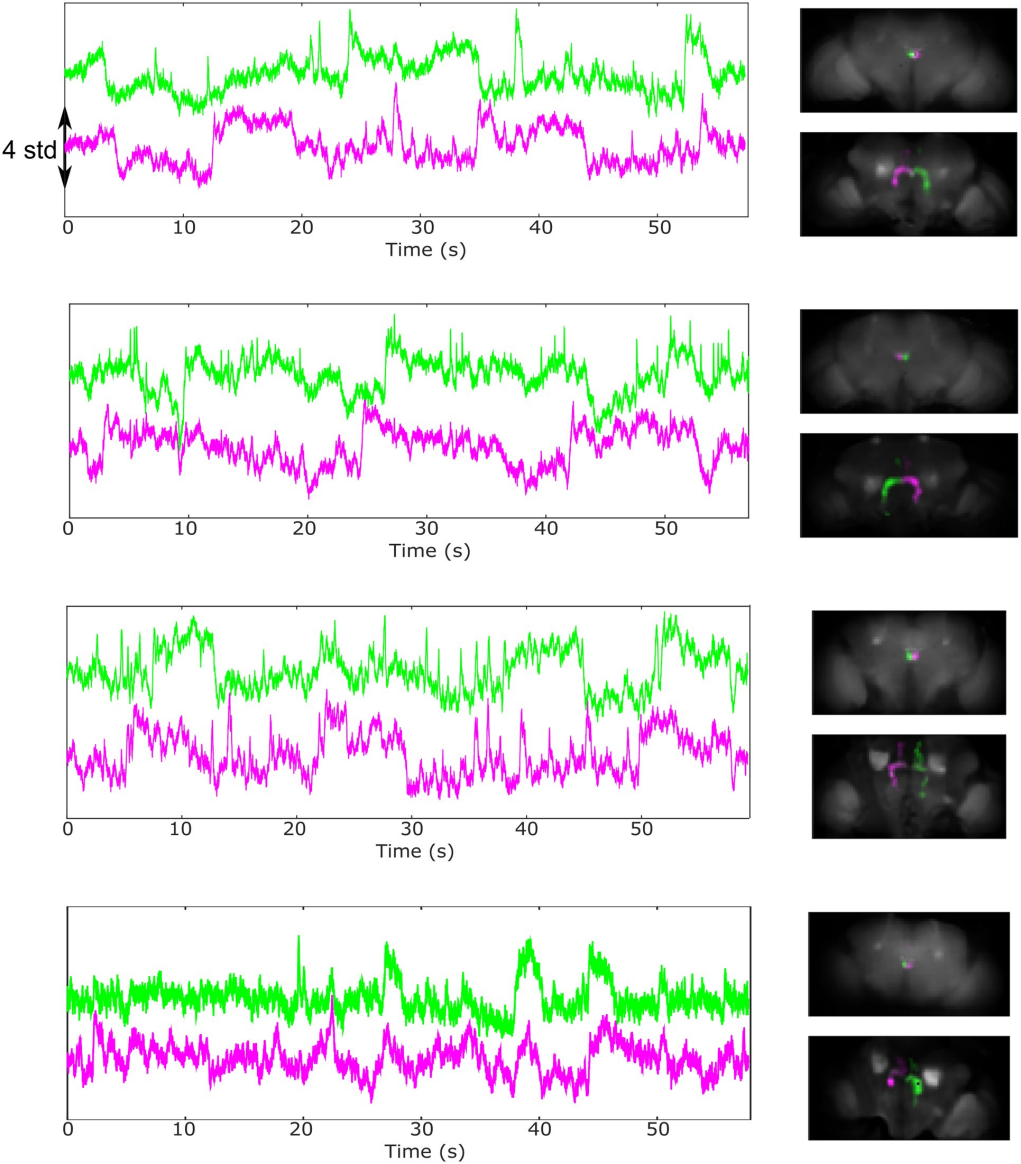
Spontaneous switches between up- and down-activity states for components in a nodulus and the contralateral part of the protocerebral bridge. We detected these components in all flies examined (genotype: *UAS_ArcLight* and from top to bottom: *nsyb-Gal4*, *nsyb-Gal4*, *Cha-Gal4*, and *GMR57C10-Gal4*, *nsyb-Gal4*). The images on the right present the two components (in different colors) at two different z planes (at the level of the noduli and fan-shaped body and at the level of the protocerebral bridge). Note that the flies were not walking in those experiments. See also S24 Fig. *Cha-Gal4*, choline acetyltransferase-Gal4; *nsyb-Gal4*, nSynaptobrevin-Gal4.

One clear difference between voltage and calcium was the presence in the ArcLight data of slow, spontaneous switches between the up and down levels of activity for components in a nodulus and contralateral part of the protocerebral bridge (Fig 8). We did not observe those components in controls in which GFP was expressed pan-neuronally. Furthermore, although some movement artifacts could generate slow fluctuations in those regions, we only observed asynchronous steps when using ArcLight (see difference between the two sides in S24 Fig). Other patterns of spontaneous activity included oscillations in the antennal lobe and lateral horn and fast, ongoing activity in the ellipsoid body and the protocerebral bridge (see S25 Fig for an experiment in which different time scales of spontaneous activity were detected). More work is necessary to establish the conditions and consistency of these patterns of activity.

Note that time series from single trials had high enough signal-to-noise ratios to detect graded potentials (e.g., components in the optic lobe in response to the onset and/or offset of flashes of light) and spike-like signals, possibly containing several action potentials (e.g., spontaneous activity and odor response for components in the antennal lobe, mushroom body, and lateral horn [Fig 7 and S25 Fig]), which are consistent with previous literature [21] [20]. Spike-like signals were particularly clear in a more restricted driver for dopamine and serotonin neurons (i.e., *TH-Gal4* and Dopa-decarboxylase or *DDC-Gal4*), as shown in S26 Fig.

### Comparison of light field imaging to other techniques for adult Drosophila large-scale imaging

Recent studies have shown that large-scale brain imaging in flies is possible with other imaging methods. Mann and colleagues [26] used a high-speed, two-photon microscope to image the brain with higher resolution but at a slower rate (1 Hz) in the absence of stimuli or behavior. We applied our analysis pipeline to these data to compare it to the results from the light field microscope (see S27 Fig). Out of a total of 23 activity-related components (compared to an average of 174 for our light field recordings, as described above), eight could be interpreted as cell bodies that could not be extracted at this resolution from the light field data. Other components—components covering the antennal lobe, mushroom body, and lateral horn; components in the pars intercerebralis; and components in the antennal lobe glomerulus, calyx, and lateral horn (likely representing activity from antennal lobe projections neurons)—had similar spatial distribution as components extracted from the light field data.

Faster techniques have also been used to image large-scale activity in flies: [27] used a Bessel beam to image 25% of the brain at 3.6 Hz, and [28] used a light sheet approach (swept confocally aligned planar excitation [SCAPE]) to image a large portion of the brain at 10 Hz. To test whether the fast-frame rate of the light field method enabled detecting signals otherwise undetectable with slower imaging methods, we subtracted the data smoothed over 100 ms, thus revealing only the activity above 10 Hz. All GCaMP6f data sets (*N* = 7) maintained some activity-related components (e.g., S28 Fig) that could be different in different flies. When we did the same analysis with ArcLight data, four out of seven flies had at least one activity-related component. Note that more information from fast activity could be present in the data, but the low signal-to-noise ratio makes it difficult to detect it with PCA/ICA.

## Discussion

In this article, we presented a method to record fast, near-whole brain activity in behaving flies, offered unique data sets produced by the method, and provided two examples of analysis pipelines that highlight this technique’s advantages.

First, we showed that the technique helps characterize global state changes related to response to stimuli and behavior. In particular, when looking at global activity in response to stimuli from different modalities, we obtained activity increases in brain regions known to be involved in processing these stimuli. Furthermore, we found a global pattern of activation when the fly walked in comparison to when it rested. In contrast, the activation was highly localized (in the areas of the AMMC, wedge, and saddle) when it groomed. This is consistent with a recent large-scale optogenetic study that reported that the manipulation of many regions throughout the brain altered walking but only a small region—the saddle—altered grooming [29]. The global activation during walking could be mediated by neuromodulators, and we indeed found that most dopamine neurons had low activity during resting or grooming but that many of these neurons, distributed over the whole brain, were strongly active during walking. Dopamine activity could affect processing of underlying circuits (e.g., changing gain [30]) but could also be important for operant learning.

Second, we used a blind source—separation algorithm to extract spatially distinct sources of activity. These sources matched anatomical subneuropil regions and thus helped identify neuron classes and sometimes a specific neuron type involved in the processing of specific stimuli or behavior but also helped detect areas that were spontaneously active in the absence of changing stimuli and behavior. For example, we extracted activity in a nodulus and opposite side of the protocerebral bridge that switched between up and down states of activity. The maps had some intensity in the lowest layers of the fan-shaped body and thus were similar to the pattern of expression of genetic drivers known to be involved in the fly’s handedness (PB-FB-No neurons from [31]) but different from neurons directly involved in turning during walk (PB-EB-No neurons from [32]). The time series resembled the flip flop pattern that was measured in the moth with intracellular recordings from neurons in the lateral accessory lobe [33], which is directly connected with the protocerebral bridge [18]. Note that the ability to record membrane voltage signals in the actual neuronal processes [34–36] performing the computation is an advantage over patch clamp experiments, which can only be recorded from the cell body at the periphery of the brain in Drosophila and thus may not be representative of the activity in the neuronal processes [37].

The data sets (available on CNCRS.org) of near-whole brain imaging and behavior contain additional, unexplored patterns of activity. More analysis and other techniques will be necessary to extract all meaningful information.

The current method has several limitations that would benefit from being addressed in future work.

### Temporal resolution

The method permits imaging of the near-whole brain with a frame rate of 200 Hz; however, the time response of the probes we used in this study is slower (rise time to spike peak of approximately 0.15 seconds for GCaMP6f and approximately 0.1 seconds for ArcLight in our hands). Fast activity can still be detected, but the probe’s response imposes a temporal filter on the underlying activity. The ability to record high signal-to-noise transients with such a high frame rate suggests that the light field microscope will be suited to measure activity from faster probes. This will help bridge the gap between the current fast local methods using microelectrodes (e.g., recording spikes and fast oscillations) and slower large-scale methods (e.g., calcium imaging).

### Effect of the excitation light

The excitation light excites the eye photoreceptors, thus affecting the fly’s ability to see as well as potentially changing brain activity states and behavior. The fly’s blue light receptors could be genetically removed and potentially replaced by another receptor, such as one for UV light, if a future researcher wanted to study brain responses to visual inputs without the strong background activation from the excitation light. For applications that do not necessitate visual inputs, blind fly mutants (e.g., a norpA, cryptochrome mutant) could be used to affect all light detection in the brain. Finally, blue-light excitation probes could be replaced with probes with longer excitation wavelengths.

### Effect of the preparation on the state of the fly

Dissection could have affected the fly’s state. Removing the cuticle on the back of the brain could affect brain activity by activating nociceptor neurons (e.g., those in the bristles). Dissection could also have affected the fly’s global health state. Indeed, we found that flies expressing ArcLight pan-neuronally were usually less active after dissection than they were before, making it difficult to obtain reliable behaviors with this genotype. Finding the optimal recovery time after dissection could help minimize these effects. Imaging the brain of nondissected flies genetically modified to have a cuticle with low absorbance (e.g., yellow flies) could also help characterize the effects of the dissection.

Although the fly could still move its legs, abdomen, and (to a limited extent) its wings, the immobilization of its head, proboscis, and thorax could have affected brain activity and behavior by imposing unnatural constraints. Furthermore, the fly’s head was tilted more than 45 degrees in comparison to its natural position in order to better align the thinner part of the brain to the z-axis. This helped minimize the loss of resolution with depth. We observed a seemingly natural behavior in this configuration (with alternations between grooming and walking as free flies do); however, we sometimes found the fly displaying unnatural behaviors, such as pushing the ball away (see behavior and corresponding brain activity in S5 Movie) or touching the holder with its legs.

Another problem resulting from immobilizing the fly’s head was the lack of coupling between the stimuli position and the fly’s movement that would normally occur in a natural setting. This problem can be solved by using a virtual reality setup in a closed loop configuration (e.g., using the movements of the ball to change the stimuli position).

### Data production rate

The whole procedure made it impractical to obtain data from a large number of flies. Even with practice, fly preparation remained challenging to the extent that it was difficult to obtain more than one good preparation per day. Another factor limiting data production—but that should be less and less of a problem as computing cost continues to drop—was the reconstruction step, which takes approximately 10 hours on a cluster of 16 graphical processing units (GPUs) for a data set of 60 GB (which corresponds to approximately 1 minute of recording at 200 Hz). This method is thus suited for studying complex spatiotemporal patterns and identifying neurons and brain structures in a few trials and flies but not for larger studies, such as genetic screens. Detecting sources from the raw light field data could help reduce the cost of reconstruction. For example, anatomical maps could be transformed back to a light field image and used as seeds for the source extraction algorithm [8].

### Detection of neuropil regions and neurons

Although we can observe the whole central brain (though the access to the gnathal ganglia depended on the quality of the preparation) as well as a large part of the optic lobes (typically the lobula and most of the medulla), we cannot observe all of the fly neurons. In particular, the ventral cord in the fly thorax is not accessible with the current setup. Imaging the ventral nerve cord in addition to the brain might be feasible with an objective with a larger field of view and the appropriate dissection preparation [38].

As the brain contains approximately 10^5^ neurons, and we record, at most, several hundred activity-related components, we are far from obtaining recordings from all neurons. This could be due to various reasons. First, some neurons might be silent and thus undetectable by our algorithm, which is based on temporal changes. Second, the signal-to-noise ratio might be insufficient for PCA and ICA to detect the activity in some processes. To increase the signal-to-noise ratio and obtain more components, future researchers could use a more sensitive probe, record longer time series, or use faster probes to obtain more temporal information. Third, several neurons might contribute to one component. Indeed, neurites with similar presynaptic inputs and thus similar activity patterns will likely have similar geometry, making them indistinguishable to the algorithm. Additionally, the resolution of the microscope is, in general, larger than individual somata and neurites. In particular, the low-axial resolution far from the focal plane (which could be improved with improved light field techniques [9]) makes it difficult to sort out the activity from regions that are close to each other, such as the antennal lobe and the lateral accessory lobe or the protocerebral bridge and the antler. However close to the focal plane, the functional maps were the same scale as functional maps obtained with higher-resolution microscopy techniques (e.g., in the fan-shaped body [17] and the lateral horn [39]) or regions known to be functional units (e.g., antennal lobe glomeruli and ellipsoid body wedges and tiles). Using a second color and complementary drivers (e.g., drivers for excitatory versus inhibitory neurons or drivers for main neurotransmitters versus drivers for neuromodulatory neurons) could increase the number of components that can be detected.

The identification of anatomical structures could also be improved. Currently, the registration of the light field data with the anatomy is done using landmark registration. This method is imprecise in brain areas that lack clear landmarks, such as the ventral areas. Concurrently imaging the brains using a different microscopy technique with higher resolution could help detect more landmarks or make it possible to use different registration techniques. Another way to improve registration would be to use driver lines for specific regions expressing a fluorophore with another color (e.g., red fluorescent protein [RFP]). Expressing activity probes with drivers for specific regions could also be used to verify the presence of components in those regions. Automating the search for matches between components’ maps and neurons in large databases such as Flycircuit or Virtual Fly Brain would help to get to the level of neurons rather than brain regions.

The maps obtained using PCA and ICA can have regions with both positive and negative values, but this study has ignored the negative parts of the maps. More work is necessary to characterize the meaning of those negative values. In particular, neurons underlying the positive part of the maps could be inhibiting the neurons underlying the negative part of the maps or vice versa.

### Time series interpretation

The PCA/ICA algorithm used here helps to unmix neural activity from movement artifacts or from other overlapping processes as well as scattered activity coming from other parts of the brain (see S12, S13, S15–S17, and S21 Figs). However, the interpretation of these time series is not straightforward, as there is no guarantee that the algorithm will extract the full neural activity from one source. Furthermore, the imperfect spatial separation of the sources can lead to artifacts in the case of strong synchronous fluorescence changes in large parts of the brain. For example, a negative signal can be present for components in the optic lobe after the odor is presented. As this signal is not present when measuring fluorescence in the region of interest delimited by the z-scored maps (putting all pixel values below three times the standard deviation to zero) or when applying PCA and ICA in the region of the optic lobes only, it is likely due to an imperfect separation of the optic lobe components from the regions in the middle of the brain where fluorescence strongly changes in response to the odor. Indeed, the maps for the optic lobe components have small negative values in the mushroom body and antennal lobe areas. To recognize these artifacts, observing both the unmixed time series and the fluorescence of ROI is thus advisable, as done in S13 and S15–S17 Figs. Using different algorithms might help prevent these artifacts. For example, non-negative matrix factorization could better separate overlapping processes and avoid spurious negative values in the maps; however, in our hands, the components were less localized and thus more difficult to interpret than with PCA/ICA.

Movement correction with 3dvolreg can be imperfect and even can, in some cases, introduce additional artifacts when strong fluorescence changes are present in large parts of the brain. Furthermore, the algorithm uses rigid registration and does not correct for local deformations. Although we partly subtract these artifacts at the PCA and ICA stages of the analysis, they can complicate the interpretation of some of the time series. Better movement-correction methods with a limited sensitivity to fluorescence changes (such as sparse and low-rank decomposition [7,40]) and nonrigid registration [41–43] as well as using an activity-independent fluorophore in another color channel as a reference would improve the reliability of the time series.

### Conclusion

Despite these limitations, the methods presented in this study can be used as a functional screen to identify brain regions and neurons involved in processing any stimulus or behavior that a fly can perform under the microscope. Furthermore, complementary to screens using activation or silencing of specific neurons, the voxels’, regions’, and components’ time series give insight into the dynamics of the network. For example, the widespread activity patterns observed here during walking suggest that coordination between different brain areas is involved. This is just the first step in exploring large-scale brain states, as this technique opens up a new window into the neural mechanisms underlying fly behavior.

## Methods

### Fly rearing and preparation for imaging

We used the GAL4/UAS system to express activity probes in neurons. The fly genotype was as described in the figure legends, and fly stocks were obtained from the Drosophila Bloomington Stock Center, Bloomington, Indiana. Flies were reared at 25 °C with a 24 hour light/dark cycle on brown food (containing cornmeal, molasses, yeast, soy flour, agar, proprionate, and nipogen), which had lower autofluorescence than yellow food (such as the one from the Bloomington Stock Center, which contains yellow cornmeal, malt extract, corn syrup, yeast, soy flour, agar, and proprionic acid).

Fly holders were 3D printed using S1 File. A piece of tape (0.75 inches wide) was shaped as a 1-mm high step using a 1-mm thick glass slide, and an aperture, as is shown in S1 Fig (1 mm wide for the body and 0.6 mm wide for the head), was made by hand using a sharpened scalpel or a thin lancet (36 gauge). The tape was then stuck onto the chamber, aligning the opening of the tape to the center of the holder. We added nail polish at the contact between the tape and the holder to avoid leaks. We also added black nail polish to the tape to block the excitation light from hitting the fly’s eyes.

Note that although the black painted tape protected the flies’ eyes from direct illumination by the microscope’s excitation light, the light scattered by the brain can still activate the eye’s receptors for blue light, as the transient activity in the first few seconds of each experiment demonstrates (see, for example, the optic lobe trace in S6 Fig). To verify that these receptors were not saturated, we presented flashes of 470-nm blue light as external stimuli (see S29 Fig). Although the stimuli excited fluorophores nonspecifically, PCA and ICA could still extract neuronal calcium responses in the optic lobes and the protocerebral bridge, thus demonstrating that the fly could still perceive external blue stimuli.

Besides activation at the onset of excitation light, we observed two types of activity patterns, likely due to the excitation light (in particular when the ommatidia were not completely protected by the black tape) but that could also be intrinsic activity (perhaps important for development). First, we observed sudden discharges in medulla columns projecting to lobula layers (for example, see Fig 7, second and third traces). Second, in some calcium recordings, we observed large oscillating waves propagating onto the medulla and along the lobula.

At the start of an experiment, flies were transferred to an empty glass vial and left on ice for approximately 1 minute. The holder was put in contact with wet tissues on ice under a stereomicroscope. A fly from the cold vial was pushed into the holder’s opening so that the posterior part of the head was in contact with the tape. UV-curing glue was added at the junction between the tape and the head between the eyes and cured for 5 seconds using a 365-nm Thorlabs LED light at 20% of power for 5 seconds. A piece of thin copper wire (wire magnet, 40 gauge) or a piece of tape was placed above the legs to push them away from the proboscis (see S1 Fig). UV glue was then added at the rim of the eye and all around the proboscis (which was pushed into the head), without touching the antenna or the legs, and was cured for 5 seconds. Uncured glue was carefully removed with tissues. A small amount of vacuum grease was placed around the neck behind the proboscis to avoid later leaks. The wire or tape was then removed, and a small piece of tissue paper or a small polystyrene foam ball was given to the fly to walk on to monitor its health during the following steps.

The holder was turned over, and the fly’s thorax was pushed down to clear the way to the back of the brain. Small pieces of tape were added onto any remaining holes around the fly’s body, and UV glue was added on top of them and cured around the thorax to fix it in place. Vacuum grease was then pushed toward the neck with a tissue. Saline (108 mM NaCl, 5 mM KCl, 3 mM CaCl2,4 mM MgCl2, 4 mM NaHCO3, 1 mM NaH2PO4, 5 mM trehalose, 10mM sucrose, 5 mM HEPES adjusted to pH 7.35 +/− 0.05 with NaOH, prepared weekly) was added and left for a few minutes to make sure that there were no leaks.

Fresh saline was added, and dissection was started with forceps that had been previously sharpened as finely as possible by hand. We first removed the cuticle in the middle of the back of the head, being careful to cut pieces before pulling them out. This exposed the hole in the middle of the brain where muscle 16 resides. The pulsatile piece was pulled out. Fresh saline was added, and the remainder of the cuticle was removed piece by piece. The brain was washed with saline several times to remove fat bodies. The air sacs were then removed very carefully as to try not to displace the brain. After a new wash with saline, the fly was ready for imaging.

### Imaging setup

The microscope was modified from an upright Olympus BX51W with a 20x NA 1.0 XLUMPlanFL or a 40x 0.8 NA LUMPLFLN objective (from Olympus). A microlens array with pitch = 125 μm and f/10 to match the 20x objective or f/25 to match the 40x objective [3] (from RPC Photonics) was positioned at the image plane using a custom-made holder (with some parts from Bioimaging Solutions, Inc.). Two relay lenses (50 mm f/1.4 NIKKOR-S Auto from Nikon) projected the image onto the sensor of a scientific CMOS camera (Hamamatsu ORCA-Flash 4.0). Note that when using half of the camera frame to attain 200 Hz for voltage recordings, the brain fit within the field of view, but rays coming from points far from the focal plane with a large angle were missed, slightly impairing reconstruction. A 490 nm LED (pE100 CoolLED) at approximately 10% of its full power was used for excitation. We used a 482/25 bandpass filter, a 495-nm dichroic beam splitter, and a 520/35 bandpass emission filter (BrightLine, Semrock) for the fluorescence. We measured the power at the sample with a power meter and found that it was up to 1 mW for the 40x objective and 4 mW for the 20x objective. Photobleaching led to a decrease in intensity after 30 seconds of 13% (*N* = 12, SD = 9%) for GCaMP6 and 20% (*N* = 6, SD = 13%) for ArcLight. Note that the full setup cost approximately US$50,000 (US$65,000 with the 64 Gb of RAM acquisition computer and the 256 Gb of RAM analysis computers), which was substantially cheaper than other cutting-edge microscopy techniques such as two-photon microscopes.

The resolution as a function of depth (see S2 Fig) was determined by imaging 2-μm fluorescent beads dispersed in an agarose gel. After reconstruction, the center of beads at different distances from the focal plane were recorded using ImageJ, and a MATLAB program measured the point spread function’s axial and lateral full width at half maximum (see https://github.com/sophie63/FlyLFM for the code).

The lateral field of view for the 20x objective was 624 x 636 square microns (312 x 309 for the 40x objective), as was determined using a mire.

The fly holder was positioned on a U-shaped stage above an air-supported ball so that the fly could walk (see Fig 1). The ball was either polyurethane foam (10 mm in diameter), Styrofoam, or hollow HDPE (one-fourth inch). We prepared a cup matching the ball diameter and with a 1.2-mm hole using self-curing rubber (from Sugru) or machining aluminum. A bottle of compressed air provided a steady flow in a pipeline consisting of a tube and a pipette tip connected to the cup hole. A micromanipulator (from Narishige) positioned the ball under the fly’s legs. For some flies, we instead provided a small Styrofoam ball that the fly could hold. The fly and the ball were illuminated by a row of IR LEDs (940 nm) in front of the fly and were observed at 100 Hz using a small camera (FFMV-03M2M from Point Grey).

To better align the behavior with the fluorescence in some experiments, the camera for monitoring the behavior and the fluorescence were synchronized by using the output of the Flash4.0 camera to trigger the acquisition from the behavior camera. When imaging fluorescence at 200 Hz, one triggering signal out of two was ignored by the slower behavior camera that recorded at 100 Hz. We recorded the fluorescence images with HCImage (from Hamamatsu) and streamed them to RAM on a 64 Gb of RAM computer, which allowed us to record approximately one continuous minute.

For the odor stimulus, air was delivered by a pump through an electrically controlled valve (12 Vdc normally closed solenoid valve), bubbled in 50% ethanol or 50% apple cider vinegar in a vial, and blown toward the fly through an inverted 1-mL pipette tip. The valve circuit was controlled by a relay connected to a LabJack U3-HV through a LJTick-RelayDriver (from LabJack). For visual stimulation, the excitation light and a 365 nm or 470 nm LED were also triggered by the LabJack. The LabJack was controlled using MATLAB programs (see https://github.com/sophie63/FlyLFM for the code).

### Analysis

We reconstructed the light field images using a program in Python, as described in [3]. Briefly, a point spread function library corresponding to the specific setup was first generated; we typically chose to reconstruct a stack of 40 layers (separated by 6 microns), with a lateral sampling distance of either 3 or 6 microns. The voltage probe’s low signal-to-noise ratio made it more difficult to detect signals with a finer sampling, so we typically reconstructed the voltage data with a lateral sampling distance of 6 microns and the calcium data with a lateral sampling of 3 microns. We reconstructed the images using 3D deconvolution on a cluster of GPUs (generously provided by the Qualcomm Institute at UCSD). Note that reconstruction using cloud computing (AWS) would cost approximately US$0.003 dollars per volume. A data set of 10,000 time steps required approximately 8 hours to reconstruct on a cluster of 15 GPUs.

We assembled the images in a Nifti file using a python routine (Tiff2niiV2 in https://github.com/sophie63/FlyLFM), inspected and cropped them in FIJI [44], and often discarded the first 5 seconds because the strong activity in response to the excitation light made it difficult to correct for movement and photobleaching. The 3D image registration function 3dvolreg [45] from AFNI was then used to correct for rigid movements. We removed the background fluorescence and the decrease in intensity from photobleaching by subtracting a signal smoothed using a box average over 15 to 30 seconds, depending on the severity of the bleaching and the length of the recording. The time series were then multiplied by −1 for ArcLight data. For denoising, we found that a Kalman filter (from https://www.mathworks.com/matlabcentral/fileexchange/26334-kalman-filter-for-noisy-movies) with a gain of 0.5 was better than a median filter over 3 points, and we used this for the data in this paper. We then applied SVD to subtract components that were most clearly related to movement; their maps contained shadows around areas with different background intensities as shown in S4 Fig. For some flies, different conditions corresponded to different recordings, which we concatenated in time after preprocessing. The reconstructed data as well as the data after preprocessing is available on the CRCNS website (https://crcns.org/NWB/Data_sets).

For early data sets (before direct synchronization of the cameras), the fluorescence and the behavior were aligned using the onset and offset of the excitation light. The small discrepancy (approximately 30 ms per minute) between the total time given by the camera for the fluorescence and the camera for the behavior was corrected linearly. The fluorescence data was then interpolated to match the behavior data using the MATLAB function Interpolate2vid.

We manually analyzed the behavior (noting the times of the behaviors or pressing different keyboard keys when we recognized different behavior using the MATLAB GUI Video_Annotate in https://github.com/sophie63/FlyLFM). We also characterized the fly’s walk by tracking the movements of the ball using FIJI’s optic flow plugin (Gaussian Window MSE).

Maps comparing the activity during rest and walking, resting and grooming, turning left and turning right, and 1 second after stimulus presentation compared to 1 second before were obtained by simply averaging the time series in each voxel for the different conditions and subtracting these maps from one another. The positive value was colored in magenta and the negative in green, thus showing which condition dominated in which voxel.

The average of the volume time series was aligned to an anatomical template (available from https://github.com/VirtualFlyBrain/DrosAdultBRAINdomains) in which the brain is segmented into regions according to the nomenclature described in [14]. The registration was performed using landmarks with ImageJ (as described in http://imagej.net/Name_Landmarks_and_Register). We marked several points in the protocerebral bridge, the tips of the mushroom body (between the alpha and alpha’ lobes), the middle of the noduli, the lateral tip of the lateral triangles, the lateral tip of the lateral horns, the center of the ellipsoid boy, the center of the antennal lobes, and the bottom part of the trachea at the level of the noduli. Although the landmarks were readily observable with the background fluorescence (see S3 Fig, for example), making a template superposing the components to the volume average helped to visually find the landmarks.

Dimensionality was then reduced by separating the volumes into slices of thickness corresponding to the point spread function height and averaging in z for each slice. The 4D data sets were typically (x, y, z, t) = 200 x 100 x 10 x 10,000 at this stage.

For source extraction, we found that using melodic [12] from the FSL package readily produced meaningful components. However, as the code was running slowly on our large data sets, we adapted it in MATLAB to parallelize some steps. A first step of SVD was used to remove the largest part of the components before calculating the pixel-wise temporal variance (without spatial filtering) that was used to normalize the data. We then reapplied SVD and plotted the log of the singular value spectrum to automatically detect the shoulder at the point with a 45° tangent. We found that although the components with the smallest variance were noise, some activity-related components were still present after the shoulder point. As such, choosing twice the number of components at the shoulder gave a good compromise between keeping activity and removing noise components. ICA was then applied to the SVD spatial components with FastICA [46] (see ICAalamelodic.m file from https://github.com/sophie63/FlyLFM). The sign was chosen so that the average of the positive side of the map was larger than the negative side. The components were then automatically sorted by brain region; after registering the standard brain to the data, we averaged the components maps in anatomically defined regions [14] using regions masks and chose the main region as the one with the highest average. We removed components corresponding to movement or noise partly automatically (removing components present in more than five regions and containing more than 200 separate objects) and partly by hand (see example of typical artifactual components in S11 Fig) using a Jupyter notebook (the notebooks corresponding to the choices made for the figures in this paper can be found at https://github.com/sophie63/FlyLFM).

To obtain the time series from regions of interest, we first made masks using the PCA/ICA maps. We calculated the standard deviation from the value in the map and set all voxels with values inferior to three times that standard deviation to zero. We then used those masks to do a weighted average of the ΔF/F time series.

The time series for turning left or right were obtained by convolving the optic flow for the ball going left or right, with a kernel corresponding to the GCaMP6 impulse response. These time series were then regressed with the components’ time series, and we inspected the maps with the strongest regression coefficients.

### Image manipulations

Fig 1B bar was added with ImageJ, and the 3D rendering was done in Icy [47], in which transparency and contrast were adjusted globally on the volume. The component’s maps were thresholded at 3x standard deviation, and only the positive part of the maps was displayed. The image contrast was then globally adjusted in ImageJ, and the figures panels were assembled in Inkscape.

### Code availability

The MATLAB and Python code for preprocessing, PCA/ICA, and sorting of the components is available at https://github.com/sophie63/FlyLFM.

## Supporting information

S1 Movie Maximum projection of pan-neuronal GCaMP6F activity, in response to odor, with the simultaneous behavior. The white square corresponds to the presentation of a puff of ethanol. Note that there is an angle of 19 degrees between the z-axis and the anterior—posterior axis. Data can be found on CNCRS.org (http://dx.doi.org/10.6080/K01J97ZN). CNCRS, Collaborative Research in Computational Neuroscience.
(AVI)

S2 Movie Maximum projection of pan-neuronal GCaMP6F activity, in response to light, with the simultaneous behavior. The white square corresponds to the presentation of flashes of UV light. Note that there is an angle of 19 degrees between the z-axis and the anterior—posterior axis. Data can be found on CNCRS.org (http://dx.doi.org/10.6080/K01J97ZN). CNCRS, Collaborative Research in Computational Neuroscience.
(AVI)

S3 Movie Maximum projection of pan-neuronal GCaMP6F activity, with the simultaneous behavior, in the absence of stimuli. Note that there is an angle of 39 degrees between the z-axis and the anterior—posterior axis. Data can be found on CNCRS.org (http://dx.doi.org/10.6080/K01J97ZN). CNCRS, Collaborative Research in Computational Neuroscience.
(AVI)

S4 Movie Maximum projection of pan-neuronal GCaMP6F activity in a *norpA* mutant background, with the simultaneous behavior, in the absence of stimuli. Note that there is an angle of 27 degrees between the z-axis and the anterior—posterior axis.
(AVI)

S5 Movie Maximum projection of GCaMP6F activity in acetylcholine neurons, with the simultaneous behavior, in the absence of stimuli. Note that there is an angle of 55 degrees between the z-axis and the anterior—posterior axis.
(AVI)

S6 Movie Maximum projection of GCaMP6F activity in dopamine neurons, with the simultaneous behavior, in the absence of stimuli. Note the global increase in many regions when the fly walks. There is an angle of 40 degrees between the z-axis and the anterior—posterior axis. Data can be found on CNCRS.org (http://dx.doi.org/10.6080/K01J97ZN). CNCRS, Collaborative Research in Computational Neuroscience.
(AVI)

S7 Movie Maximum projection of GCaMP6F activity in dopamine and serotonin neurons, with the simultaneous behavior, in the absence of stimuli. Note that there is an angle of 60 degrees between the z-axis and the anterior—posterior axis. Data can be found on CNCRS.org (http://dx.doi.org/10.6080/K01J97ZN). CNCRS, Collaborative Research in Computational Neuroscience.
(AVI)

S1 Fig Fly preparation for imaging. The head is fixed on a black painted tape while the legs are held down with a thin wire. The chamber is then flipped over and the cuticle on the back of the brain is removed.
(TIF)

S2 Fig Resolution as a function of distance to the focal plane (FWHM). The point spread function was measured using 2-μm fluorescent beads embedded in an agarose gel. See also Fig 5 in [3]. The underlying numerical values can be found in S1 Data. FWHM, full width at half maximum.
(TIF)

S3 Fig Z-stack of ArcLight baseline fluorescence showing anatomical structures. Both A and B were acquired in 5 ms. A) 20x NA = 1.0, Bar = 90 μm. B) 40x NA = 0.8, Bar = 60 μm. NA, numerical aperture.
(TIF)

S4 Fig Schematics of the analysis pipeline. (TIF)

S5 Fig Anterior, middle, and posterior slices of activity maps for various conditions (additional flies from Fig 2). Different panels correspond to different flies. All the data was recorded with a 20x NA = 1.0 objective unless noted otherwise. A) Comparison of fluorescence intensity when the fly rests and when it is active (either walk or groom). The pixel value is green if the fluorescence is in average higher during the behavior than during rest and magenta if the fluorescence is in average higher during rest than during the behavior. Note that some flies were walking but not grooming (or grooming too little to extract the grooming activity map). B) Comparison of fluorescence intensity when the fly turns left or right. The pixel value is green if the fluorescence is higher during turning left and magenta if the fluorescence is higher during turning right. C) Comparison of fluorescence between response to stimuli and baseline activity (magenta, odor; green, light), for both calcium (GCaMP6) and voltage probes (ArcLight). Data can be found on CNCRS.org (http://dx.doi.org/10.6080/K01J97ZN). CNCRS, Collaborative Research in Computational Neuroscience; NA, numerical aperture.
(TIF)

S6 Fig Average of voxels time series (variance normalized) in large brain regions during stimuli presentation and behavior. Data can be found on CNCRS.org (http://dx.doi.org/10.6080/K01J97ZN). AL, antennal lobe; CNCRS, Collaborative Research in Computational Neuroscience; CX, central complex; GNG, gnathal ganglia; INP, inferior neuropils; LH, lateral horn; LX, lateral complex; MB, mushroom body; OL, optic lobe; PENP, periesophageal neuropils; SNP, superior neuropils; VMNP, ventromedial neuropils; VLNP, ventrolateral neuropils.
(TIF)

S7 Fig Average pan-neuronal GCaMP6F fluorescence difference between stimulus presentation (during the first second) and baseline. Different points correspond to different flies (*N* = 5). Squares represent the medians. The underlying numerical values can be found in S1 Data. AL, antennal lobe; CX, central complex; GNG, gnathal ganglia; INP, inferior neuropils; LH, lateral horn; LX, lateral complex; MB, mushroom body; OL, optic lobe; PENP, periesophageal neuropils; SNP, superior neuropils; VMNP, ventromedial neuropils; VLNP, ventrolateral neuropils.
(TIF)

S8 Fig Average pan-neuronal GCaMP6F fluorescence difference between behavior and rest. Different points correspond to different flies (*N* = 5 for walk and *N* = 4 for groom). The squares correspond to the medians. The underlying numerical values can be found in S1 Data. AL, antennal lobe; CX, central complex; GNG, gnathal ganglia; INP, inferior neuropils; LH, lateral horn; LX, lateral complex; MB, mushroom body; OL, optic lobe; PENP, periesophageal neuropils; SNP, superior neuropils; VMNP, ventromedial neuropils; VLNP, ventrolateral neuropils.
(TIF)

S9 Fig Average of pan-neuronal activity in small regions. Columns show Z-stack (from the most anterior slice of the brain presented on top to the most posterior on the bottom) of the template brain. A) Average fluorescence difference between behavior and rest (*N* = 4). B) Average of the absolute difference between turning left and right (*N* = 6). C) Average of pan-neuronal GCaMP6F or ArcLight fluorescence difference between stimulus response and baseline (*N* = 7 for GCaMP6 and *N* = 6 for ArcLight). Note that the flash of light was presented from the side.
(TIF)

S10 Fig Z-stack of the 3D maps for all components (including both activity-related and artifactual components) obtained with the PCA/ICA algorithm. Flies expressed GCaMP6F, and data were acquired with a 20x 1.0 NA objective. The maps were aligned to an anatomical template. Each column corresponds to a different fly, and rows correspond to different depth (6 μm apart) from the most anterior to the most posterior slice. Different colors were assigned randomly to different components. All columns are for pan-neuronal data except for the last column, which corresponds to a *Cha-Gal4* line. Data can be found on CNCRS.org (http://dx.doi.org/10.6080/K01J97ZN). *Cha-Gal4*, choline acetyltransferase-Gal4; CNCRS, Collaborative Research in Computational Neuroscience; ICA, independent component analysis; NA, numerical aperture; PCA, principal component analysis.
(TIF)

S11 Fig All components (including both activity-related and artifactual components) obtained with the PCA/ICA algorithm from pan-neuronal GCaMP6 data acquired with a 40x 0.8 NA objective. The maps were aligned to an anatomical template. Note that the field of view only encompasses the central part of the brain. Each column shows a Z-stack of the 3D map for different flies, and rows correspond to different depth (layers are 6 μm apart) from the most anterior to the most posterior slice. Colors were assigned randomly to different components. Data can be found on CNCRS.org (http://dx.doi.org/10.6080/K01J97ZN). CNCRS, Collaborative Research in Computational Neuroscience; ICA, independent component analysis; NA, numerical aperture; PCA, principal component analysis.
(TIF)

S12 Fig Example of common artifactual components also present in the GFP control. A) Movement artifacts: those components are present on the edge of the brain, and the time series have large transient uncharacteristic of activity probes. B) Horizontal lines resulting from aliasing after reconstruction. C) Background noise. GFP, green fluorescent protein.
(TIF)

S13 Fig Z-stack of thresholded ICA map (displayed as a heatmap overlaid on top of the background fluorescence) and time series for a few hand-selected components with varying degrees of difference between ROI average and PCA/ICA time series (same fly for S13 to S17). Red curve is the average of the fluorescence in the thresholded map, blue curve is the time series resulting from PCA/ICA, green dashes corresponds to the fly walking, cyan dashes to the presentation of odor puffs, and magenta dashes to the presentation of flashes of light. For components’ map with a thin track (A and B), possibly representing single neurons, PCA/ICA unmixes action potential-like signals from the background. For maps representing neuropil regions (such as a layer in the fan-Shaped body in C), ROI time series and PCA/ICA time series are very similar. Data can be found on CNCRS.org (http://dx.doi.org/10.6080/K01J97ZN). CNCRS, Collaborative Research in Computational Neuroscience; ICA, independent component analysis; PCA, principal component analysis; ROI, region of interest.
(TIF)

S14 Fig A) Maps’ z-stack of components correlated with the onset of UV light (magenta) or Odor (green). B) Number of components per brain region (normalized by the number for the region with the most components) that were correlated with the onset of UV light or the onset of odor (with correlation coefficient > 0.1) for flies expressing GCaMP6 pan-neuronally. Different points correspond to different flies (*N* = 5), and squares show the medians. C) Histogram of correlation coefficients (square root of R^2^) with odor onset convolved with a GCaMP6 kernel, for components present in all the three regions: antennal lobe, mushroom body calyx, and lateral horn, thus likely representing the activity of antennal lobe projection neurons. The underlying numerical values can be found in S1 Data. Data can also be found on CNCRS.org (http://dx.doi.org/10.6080/K01J97ZN). AL, antennal lobe; CNCRS, Collaborative Research in Computational Neuroscience; CX, central complex; GCaMP6F; GNG, gnathal ganglia; INP, inferior neuropils; LH, lateral horn; LX, lateral complex; MB, mushroom body; OL, optic lobe; PENP, periesophageal neuropils; SNP, superior neuropils; VMNP, ventromedial neuropils; VLNP, ventrolateral neuropils.
(TIF)

S15 Fig Z-stack of thresholded ICA map (displayed as a heatmaps overlaid on top of the background fluorescence) and time series for a few hand-selected components strongly correlated with flashes of UV light (same fly for S13 to S17). Red curve is the average of the fluorescence in the thresholded map, blue curve is the time series resulting from PCA/ICA, green dashes corresponds to the fly walking, cyan dashes to the presentation of odor puffs, and magenta dashes to the presentation of flashes of light. In C, note how the background transient corresponding to the first odor presentation is removed by PCA/ICA. ICA, independent component analysis; PCA, principal component analysis.
(TIF)

S16 Fig Detailed z-stack of thresholded heatmaps and time series for a few hand-selected components strongly correlated with odor presentation (same fly for S13 to S17). Red curve is the average of the fluorescence in the thresholded map (ROI), blue curve is the time series resulting from PCA/ICA, green dashes corresponds to the fly walking, magenta dashes to the presentation of flashes of light, and cyan dashes to the presentation of odor puffs. Note how background transients associated with the response to light (mainly above the first magenta dash) present in the ROI average are excluded from the components time series. Data can be found on CNCRS.org (http://dx.doi.org/10.6080/K01J97ZN). CNCRS, Collaborative Research in Computational Neuroscience; ICA, independent component analysis; PCA, principal component analysis; ROI, region of interest.
(TIF)

S17 Fig Detailed z-stack of thresholded heatmaps and time series for a few hand-selected components strongly correlated with walking (same fly as S13 to S16). Red curve is the average of the fluorescence in the thresholded map (ROI), blue curve is the time series resulting from PCA/ICA, green dashes corresponds to the fly walking, magenta dashes to the presentation of flashes of light, and cyan dashes to the presentation of odor puffs. Note how the background transients associated with the response to odor in the ROI average are excluded from components time series. Data can be found on CNCRS.org (http://dx.doi.org/10.6080/K01J97ZN). CNCRS, Collaborative Research in Computational Neuroscience; ICA, independent component analysis; PCA, principal component analysis; ROI, region of interest.
(TIF)

S18 Fig Components most correlated with turning left or right, using an acetylcholine neurons line (*Cha-Gal4* and *UAS-GCaMP6F*). A) Anterior, medial and posterior slices of the components most correlated to turning left (cyan and blue) and right (red and yellow). The arrows point to characteristic shapes (inverted V or fine claw-like dorsal neurites). B) Example time series (variance normalized) for the four different components along with turning behavior. Top panel shows data from a pan-neuronal line (*nsyb-Gal4*, *UAS-GCaMP6F*), and bottom panel, from the same fly as A). In each panel, the top two traces (green and blue) are proportional to angular speed of the ball towards left or right, convolved with a GCaMP6 kernel. The four bottom traces show activity components (same color code as the maps). Note that the cyan (or yellow) trace is slightly delayed compared to the blue trace (or red trace respectively). *Cha-Gal4*, choline acetyltransferase-Gal4; *nsyb-Gal4*, nSynaptobrevin-Gal4.
(TIF)

S19 Fig Additional components from flies expressing GCaMP6F in dopamine neurons (*TH-Gal4*, *UAS- GCaMP6F*). Left: components’ time series (variance normalized), right: components’ map. A and B are different flies. In A, the green dashes correspond to the fly walking. *TH-Gal4*, tyrosine hydroxylase-Gal4.
(TIF)

S20 Fig All components (including both activity-related and artifactual components) obtained with the PCA/ICA algorithm from pan-neuronal ArcLight data acquired with a 20x 1.0 NA objective. The maps were aligned to an anatomical template. Each column presents a z-stack of the 3D map for one fly. Each column corresponds to a different fly, and rows correspond to different depth (6 μm apart), from the most anterior slice at the top to the most posterior at the bottom. Colors were assigned randomly to different components. Data can be found on CNCRS.org (http://dx.doi.org/10.6080/K01J97ZN). CNCRS, Collaborative Research in Computational Neuroscience; NA, numerical aperture; ICA, independent component analysis; PCA, principal component analysis.
(TIF)

S21 Fig Separation of activity-related and artifactual components in an extreme case of strong movement and low signal to noise ratio (corresponding to column 4 of S20 Fig). Left is a z-stack of activity-related components and right are artifactual components.
(TIF)

S22 Fig Number of components per large brain region for GCaMP6 or ArcLight. In all experiments, puffs of odor and flashes of UV light were presented to the flies (*N* = 6 for ArcLight and *N* = 5 for GCaMP6). Dots are for single flies and the squares represent the medians. The underlying numerical values can be found in S1 Data. AL, antennal lobe; CX, central complex; GNG, gnathal ganglia; INP, inferior neuropils; LH, lateral horn; LX, lateral complex; MB, mushroom body; OL, optic lobe; PENP, periesophageal neuropils; SNP, superior neuropils; VMNP, ventromedial neuropils; VLNP, ventrolateral neuropils.
(TIF)

S23 Fig Number of components per brain region (normalized by the number for the region with the most components) responding to UV light or odor, for pan-neuronally expressed ArcLight. Different points correspond to different flies (*N* = 6), and squares indicate the medians. The underlying numerical values can be found in S1 Data. AL, antennal lobe; CX, central complex; GNG, gnathal ganglia; INP, inferior neuropils; LH, lateral horn; LX, lateral complex; MB, mushroom body; OL, optic lobe; PENP, periesophageal neuropils; SNP, superior neuropils; VMNP, ventromedial neuropils; VLNP, ventrolateral neuropils.
(TIF)

S24 Fig Left and right difference of variance normalized time series for nodulus and protocerebral bridge from the other side. Because no such components were automatically extracted with pan-neuronally expressed GFP, we created masks of one nodulus and the opposite side of the protocerebral bridge using an anatomical template for the GFP control. GFP, green fluorescent protein.
(TIF)

S25 Fig Pan-neuronal voltage recording with a different pan-neuronal line from the one used in Fig 7 (*GMR57C10-Gal4*, *Cha-Gal4*, *UAS-ArcLight*). Left are the component’s time series (variance normalized) and right are the corresponding maps. Note the slow oscillations in the antennal lobe and lateral horn (peak frequency of the average power spectrum smoothed over 50 points is 1.6 Hz). The cumulative power spectrum reaches 80% at 3.6 Hz for those components but only at 8.6 Hz for components with faster ongoing activity such as the first and fourth components, in the protocerebral bridge, and the ellipsoid body. Data can be found on CNCRS.org (http://dx.doi.org/10.6080/K01J97ZN). *Cha-Gal4*, choline acetyltransferase-Gal4; CNCRS, Collaborative Research in Computational Neuroscience.
(TIF)

S26 Fig Components extracted from two flies (A and B) expressing a voltage probe (with *UAS-ArcLight*) in dopamine and serotonin neurons (*TH-Gal4*, *DDC-Gal4*). Left are the component’s variance normalized time series (magenta dashes correspond to the presentation of a flash of light, cyan dashes to puffs of odor, and forest green traces to walking speed) and right are the corresponding z-projected maps. In A), for example, the first two time series correspond to the first image; the next three time series to the second image. In B), each time series corresponds to one map showed on the right. *DDC-Gal4*, Dopa decarboxylase-Gal4; *TH-Gal4*, tyrosine hydroxylase-Gal4.
(TIF)

S27 Fig PCA/ICA analysis pipeline applied to two-photon data from [26]. Different panels present a maximum z projection for different types of components as described in the text, and in each panel different colors correspond to different individual components. ICA, independent component analysis; PCA, principal component analysis.
(TIF)

S28 Fig Z-scored components maps (A) and variance normalized time series (B) from pan-neuronal GCaMP6F (*UAS-GCaMP6F*, *Cha-Gal4*, and *GMR57C10-Gal4*) data in which we subtracted the data smoothed over 100ms to keep only the activity over 10 Hz. Magenta dashes correspond to flashes of UV light and cyan dashes to puffs of odors. *Cha-Gal4*, choline acetyltransferase-Gal4.
(TIF)

S29 Fig Response to flashes of 470 nm light (*nsyb-GAL4*, *UAS-GCaMP6s*). Only the components responding to the stimulus are included. Left are the component’s variance normalized time series and right are the corresponding z-projected maps. Blue dashes correspond to flashes of blue light. *nsyb-Gal4*, nSynaptobrevin-Gal4.
(TIF)

S1 Data Data for S2, S7, S8, S14B and S14C, S22, and S23 Figs. (XLSX)

S1 File Chamber for mounting and imaging the fly. (STL)

## Abbreviations

AMMC: antennal mechanosensory and motor center
CNCRS: Collaborative Research in Computational Neuroscience
DDC: Dopa decarboxylase
fMRI: functional magnetic resonance imaging
GFP: green fluorescent protein
GPU: graphical processing unit
ICA: independent component analysis
NA: numerical aperture
PCA: principal component analysis
RFP: red fluorescent protein
ROI: region of interest
SCAPE: swept confocally aligned planar excitation
sCMOS: scientific complementary metal-oxide-semiconductor
TH: tyrosine hydroxylase
