# Supplementary figures and images for: Fast near-whole–brain imaging in adult Drosophila during responses to stimuli and behavior
Source: PLoS Biol. 2019 Feb 15;17(2):e2006732. doi: 10.1371/journal.pbio.2006732 (PMC6395010; doi:10.1371/journal.pbio.2006732)

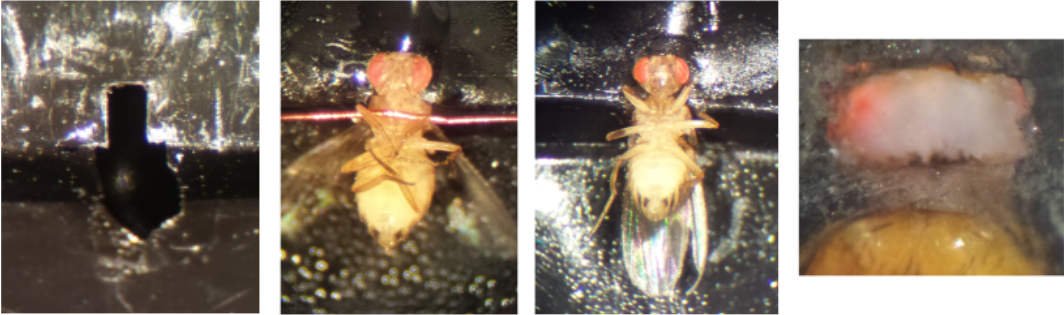

Supplement: S1 Fig — The head is fixed on a black painted tape while the legs are held down with a thin wire. The chamber is then flipped over and the cuticle on the back of the brain is removed. (TIF) [file pbio.2006732.s008.tif]

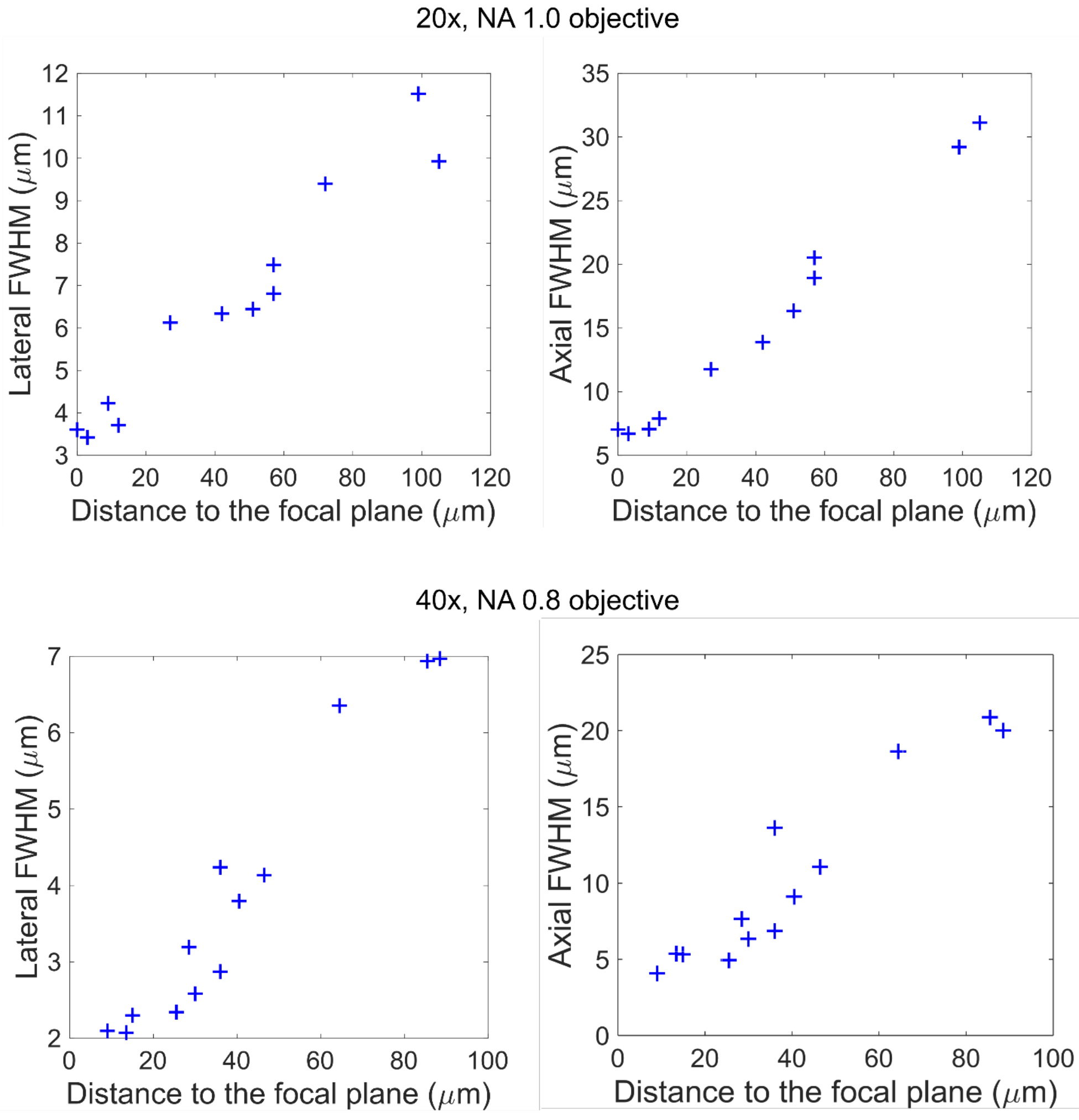

Supplement: S2 Fig — The point spread function was measured using 2-μm fluorescent beads embedded in an agarose gel. See also Fig 5 in [3]. The underlying numerical values can be found in S1 Data. FWHM, full width at half maximum. (TIF) [file pbio.2006732.s009.tif]

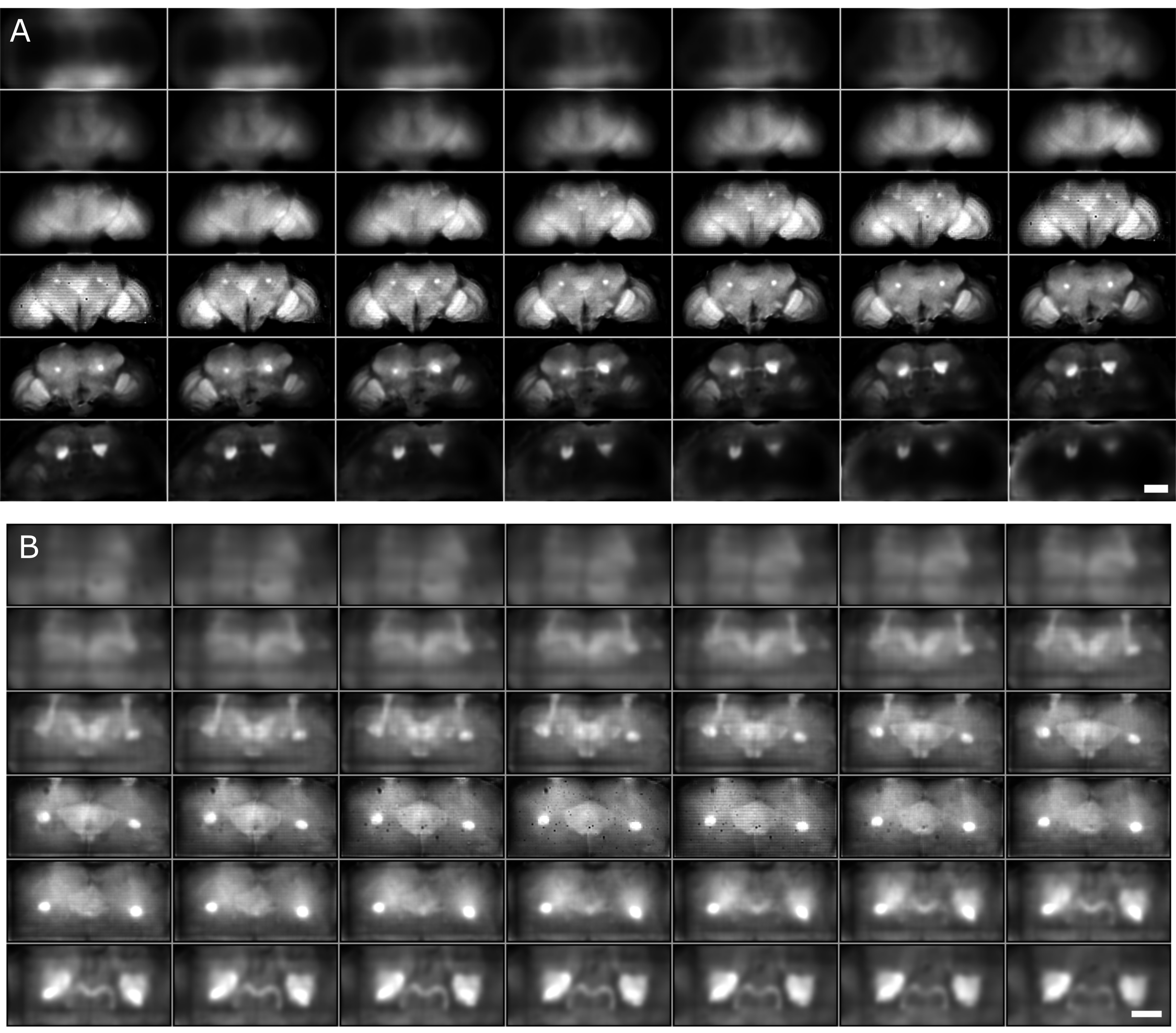

Supplement: S3 Fig — Both A and B were acquired in 5 ms. A) 20x NA = 1.0, Bar = 90 μm. B) 40x NA = 0.8, Bar = 60 μm. NA, numerical aperture. (TIF) [file pbio.2006732.s010.tif]

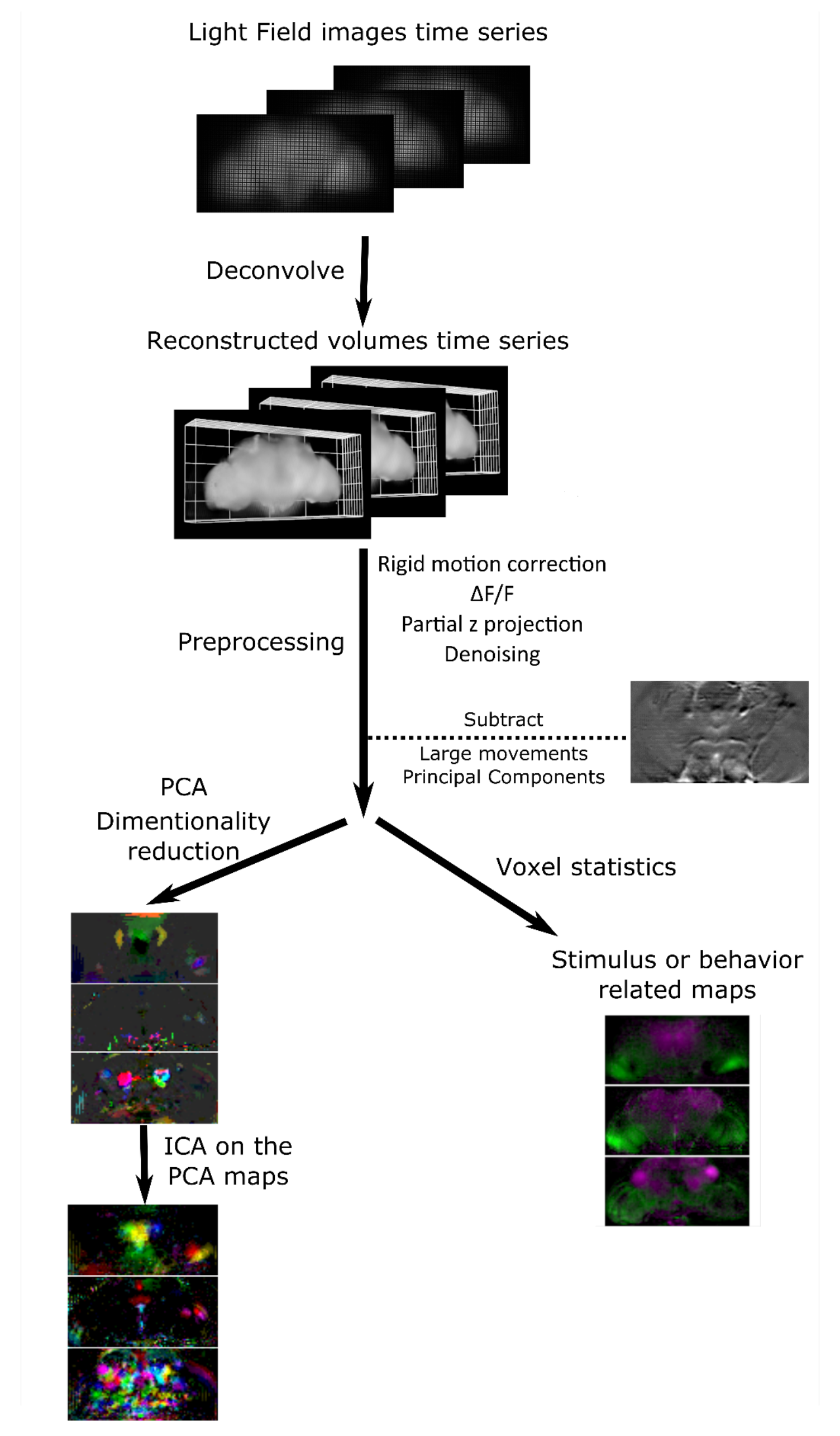

Supplement: S4 Fig — (TIF) [file pbio.2006732.s011.tif]

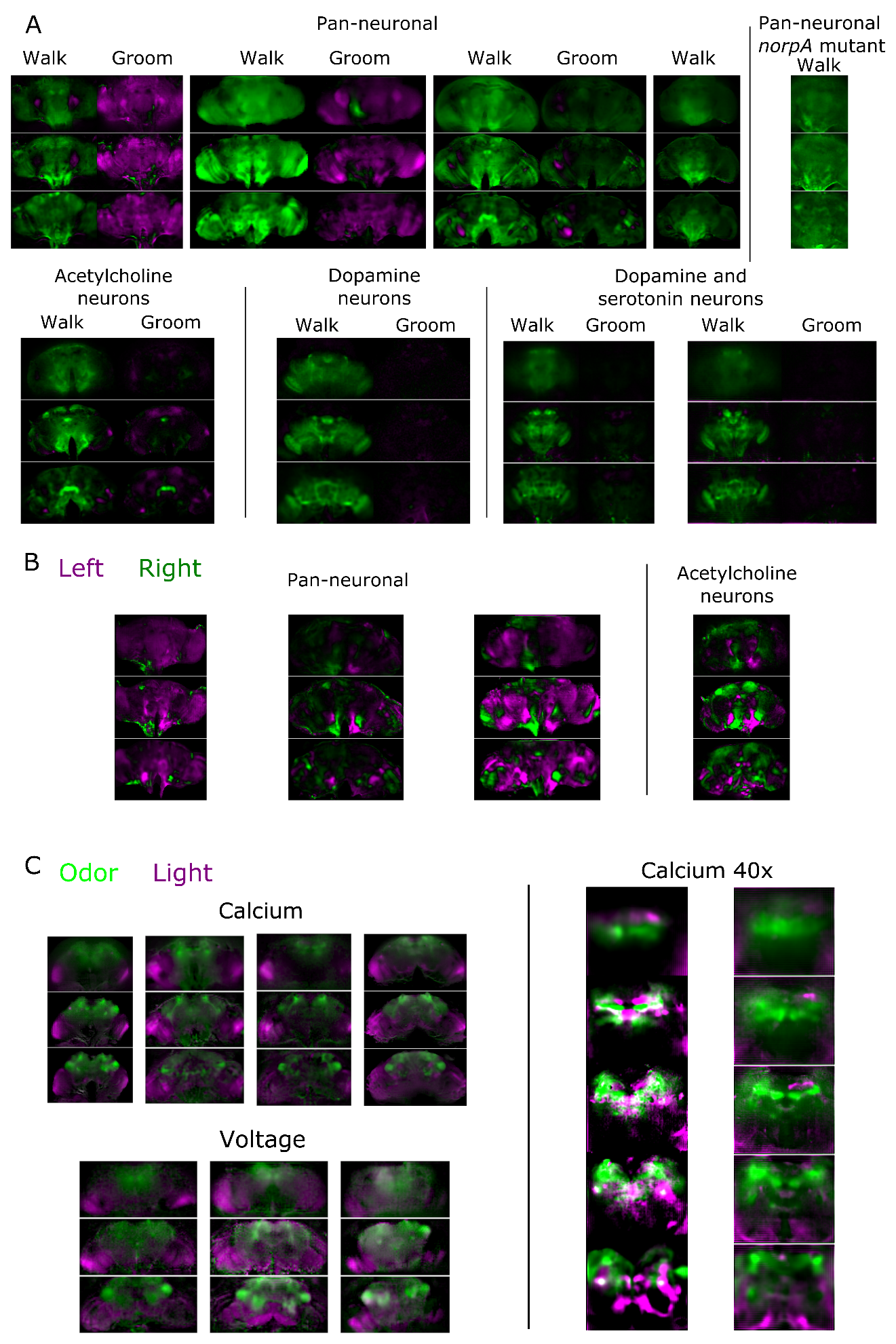

Supplement: S5 Fig — Different panels correspond to different flies. All the data was recorded with a 20x NA = 1.0 objective unless noted otherwise. A) Comparison of fluorescence intensity when the fly rests and when it is active (either walk or groom). The pixel value is green if the fluorescence is in average higher during the behavior than during rest and magenta if the fluorescence is in average higher during rest than during the behavior. Note that some flies were walking but not grooming (or grooming too little to extract the grooming activity map). B) Comparison of fluorescence intensity when the fly turns left or right. The pixel value is green if the fluorescence is higher during turning left and magenta if the fluorescence is higher during turning right. C) Comparison of fluorescence between response to stimuli and baseline activity (magenta, odor; green, light), for both calcium (GCaMP6) and voltage probes (ArcLight). Data can be found on CNCRS.org (http://dx.doi.org/10.6080/K01J97ZN). CNCRS, Collaborative Research in Computational Neuroscience; NA, numerical aperture. (TIF) [file pbio.2006732.s012.tif]

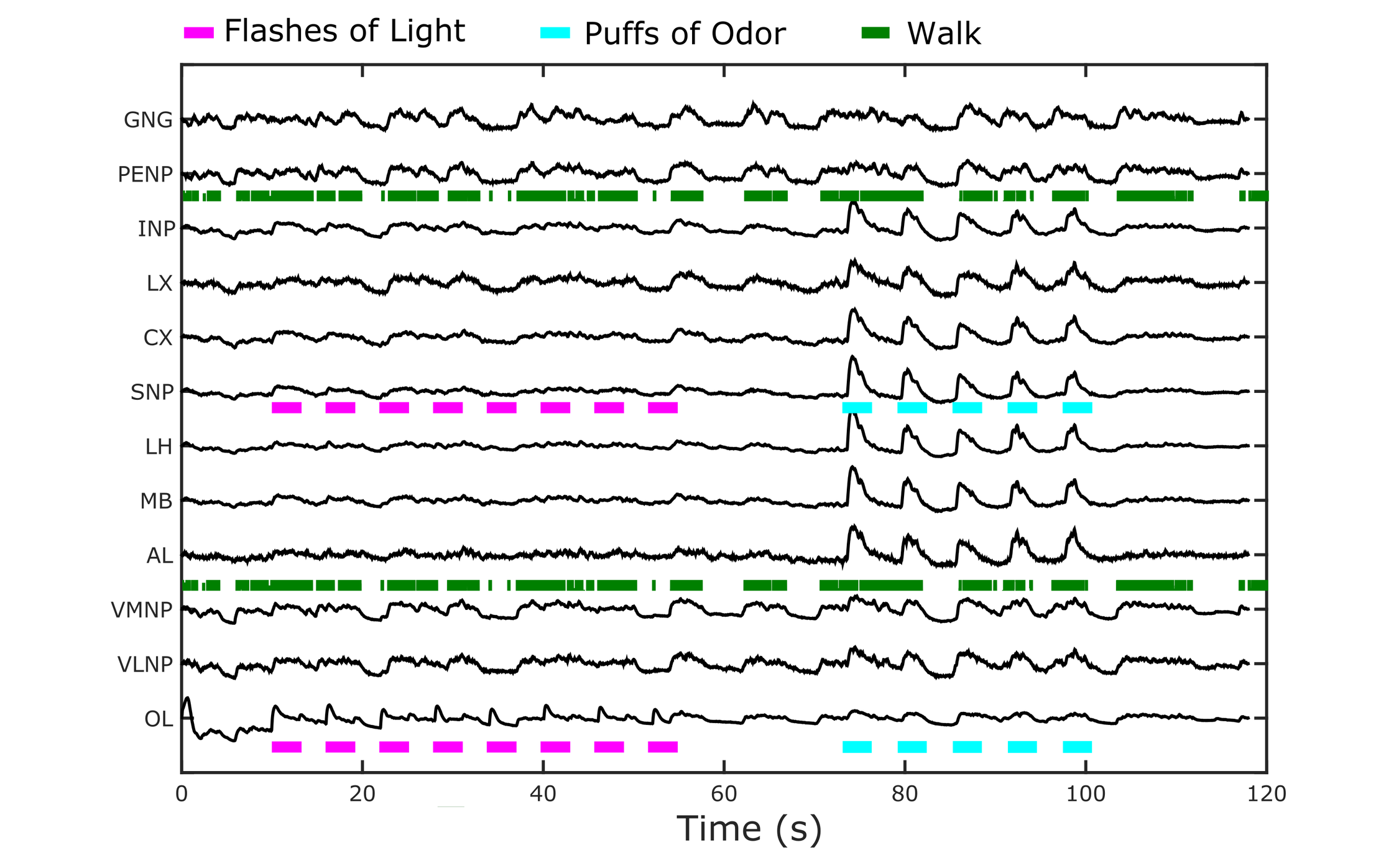

Supplement: S6 Fig — Data can be found on CNCRS.org (http://dx.doi.org/10.6080/K01J97ZN). AL, antennal lobe; CNCRS, Collaborative Research in Computational Neuroscience; CX, central complex; GNG, gnathal ganglia; INP, inferior neuropils; LH, lateral horn; LX, lateral complex; MB, mushroom body; OL, optic lobe; PENP, periesophageal neuropils; SNP, superior neuropils; VMNP, ventromedial neuropils; VLNP, ventrolateral neuropils. (TIF) [file pbio.2006732.s013.tif]

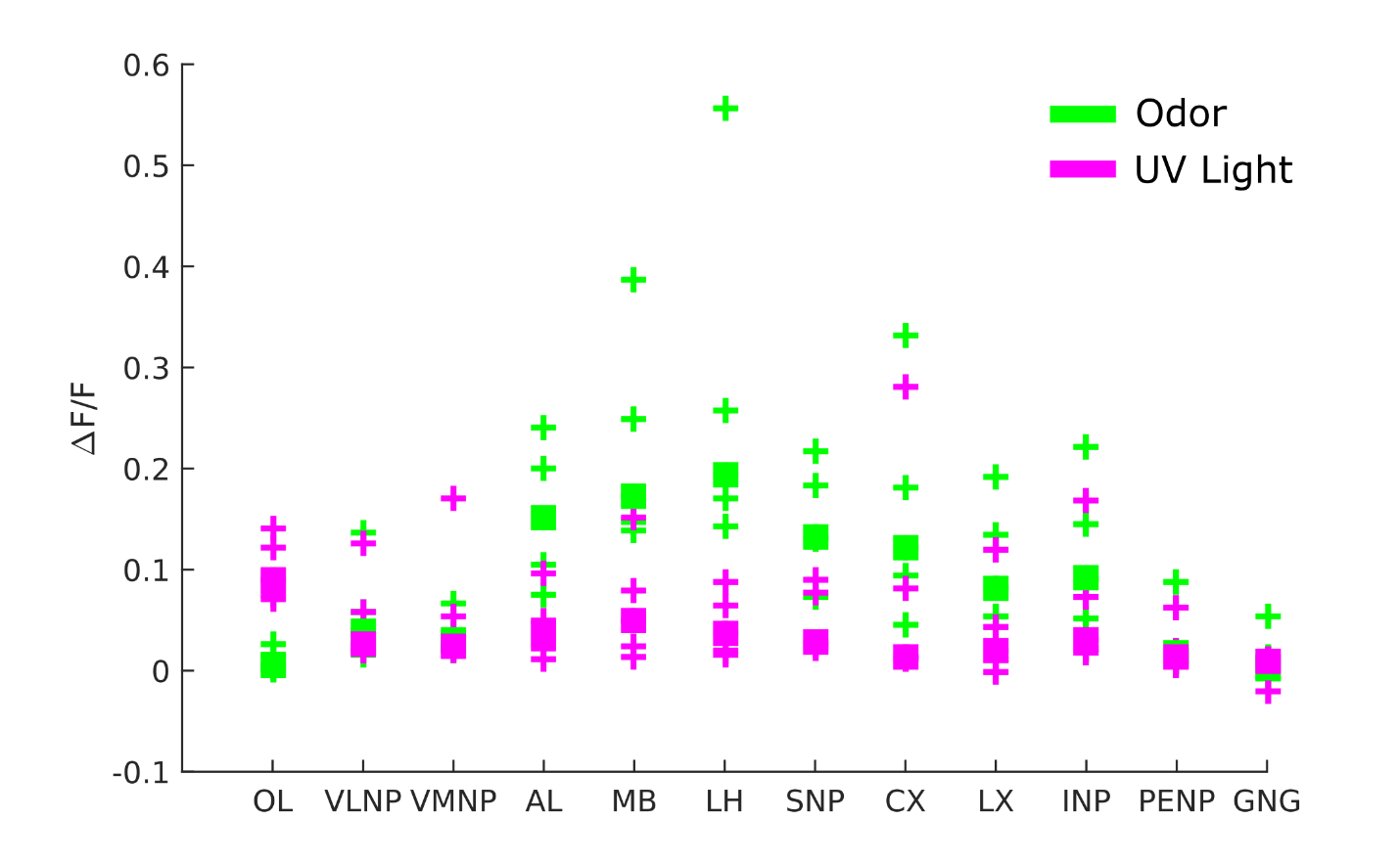

Supplement: S7 Fig — Different points correspond to different flies (N = 5). Squares represent the medians. The underlying numerical values can be found in S1 Data. AL, antennal lobe; CX, central complex; GNG, gnathal ganglia; INP, inferior neuropils; LH, lateral horn; LX, lateral complex; MB, mushroom body; OL, optic lobe; PENP, periesophageal neuropils; SNP, superior neuropils; VMNP, ventromedial neuropils; VLNP, ventrolateral neuropils. (TIF) [file pbio.2006732.s014.tif]

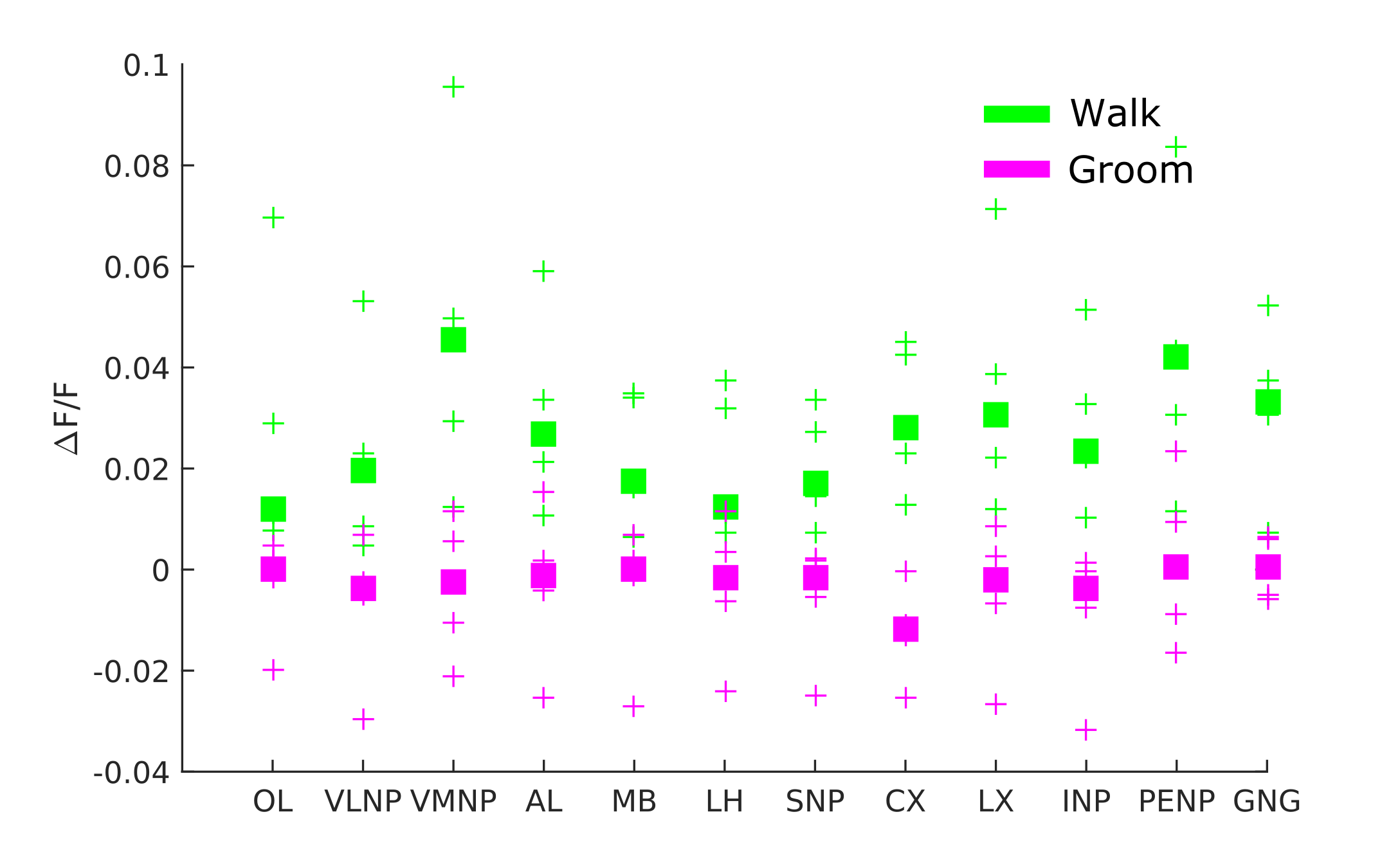

Supplement: S8 Fig — Different points correspond to different flies (N = 5 for walk and N = 4 for groom). The squares correspond to the medians. The underlying numerical values can be found in S1 Data. AL, antennal lobe; CX, central complex; GNG, gnathal ganglia; INP, inferior neuropils; LH, lateral horn; LX, lateral complex; MB, mushroom body; OL, optic lobe; PENP, periesophageal neuropils; SNP, superior neuropils; VMNP, ventromedial neuropils; VLNP, ventrolateral neuropils. (TIF) [file pbio.2006732.s015.tif]

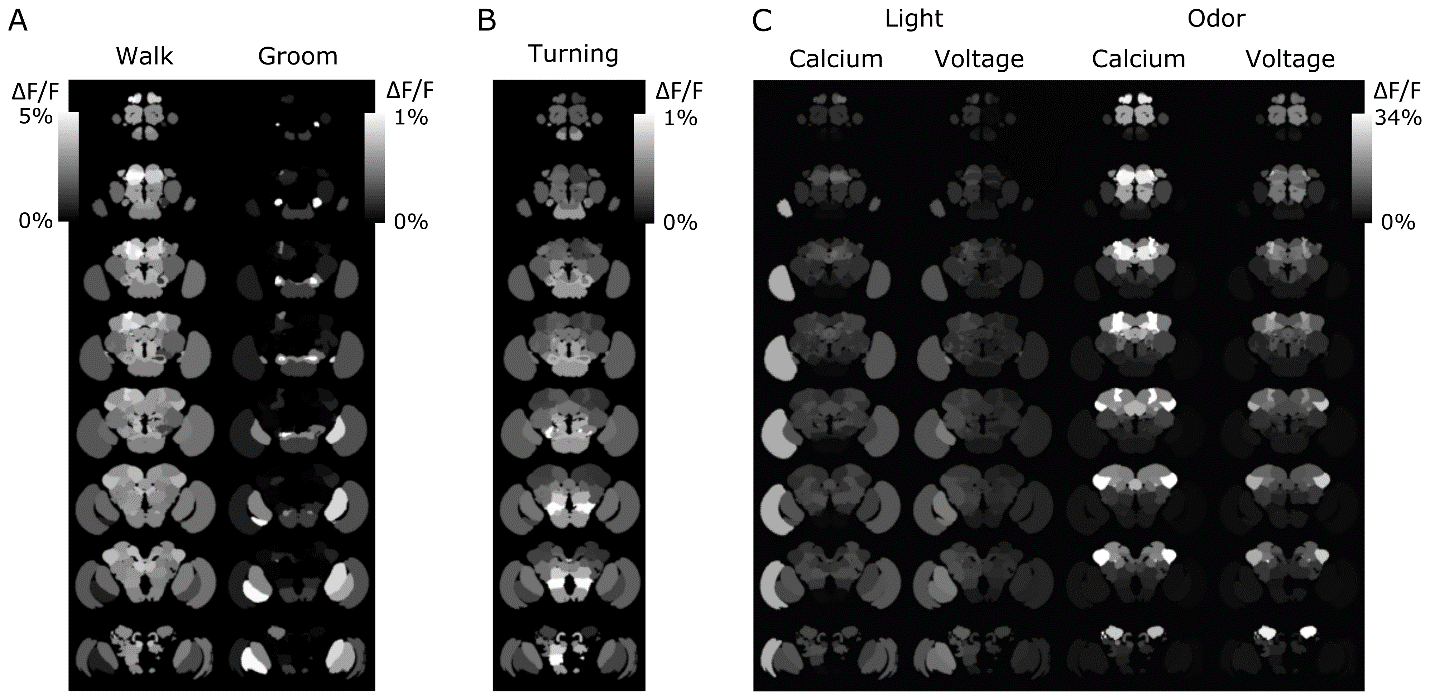

Supplement: S9 Fig — Columns show Z-stack (from the most anterior slice of the brain presented on top to the most posterior on the bottom) of the template brain. A) Average fluorescence difference between behavior and rest (N = 4). B) Average of the absolute difference between turning left and right (N = 6). C) Average of pan-neuronal GCaMP6F or ArcLight fluorescence difference between stimulus response and baseline (N = 7 for GCaMP6 and N = 6 for ArcLight). Note that the flash of light was presented from the side. (TIF) [file pbio.2006732.s016.tif]

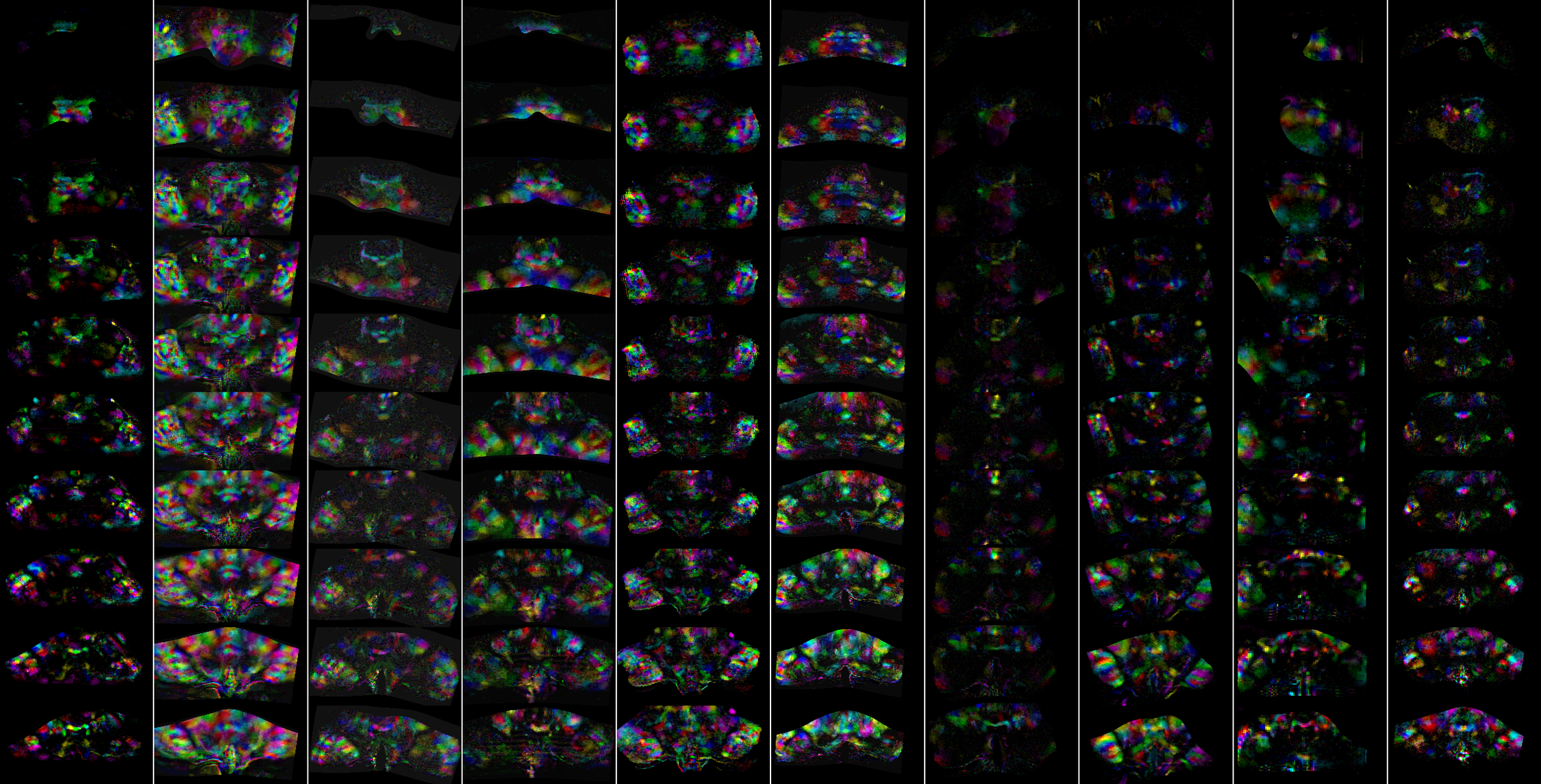

Supplement: S10 Fig — Flies expressed GCaMP6F, and data were acquired with a 20x 1.0 NA objective. The maps were aligned to an anatomical template. Each column corresponds to a different fly, and rows correspond to different depth (6 μm apart) from the most anterior to the most posterior slice. Different colors were assigned randomly to different components. All columns are for pan-neuronal data except for the last column, which corresponds to a Cha-Gal4 line. Data can be found on CNCRS.org (http://dx.doi.org/10.6080/K01J97ZN). Cha-Gal4, choline acetyltransferase-Gal4; CNCRS, Collaborative Research in Computational Neuroscience; ICA, independent component analysis; NA, numerical aperture; PCA, principal component analysis. (TIF) [file pbio.2006732.s017.tif]

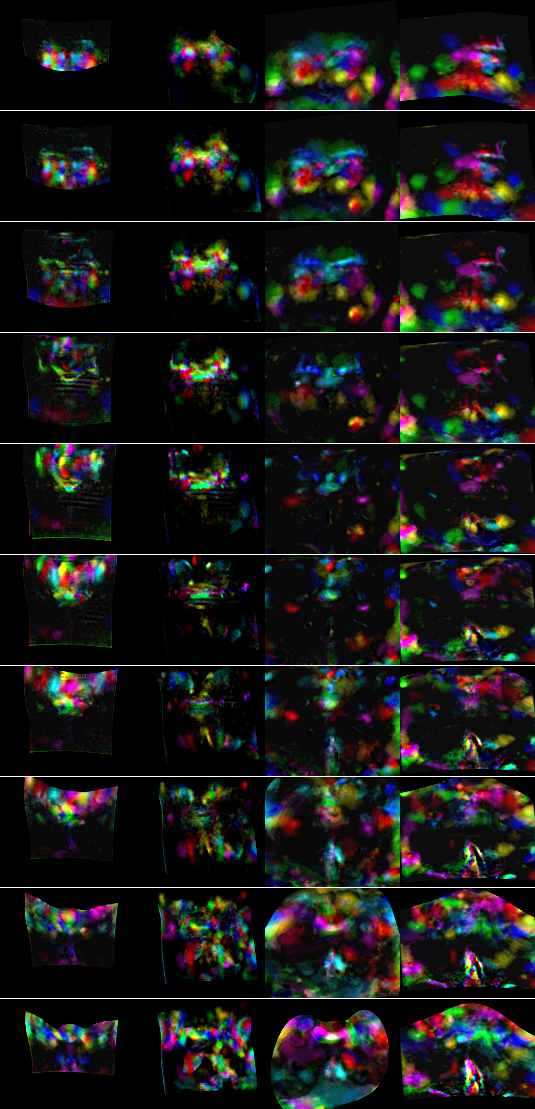

Supplement: S11 Fig — The maps were aligned to an anatomical template. Note that the field of view only encompasses the central part of the brain. Each column shows a Z-stack of the 3D map for different flies, and rows correspond to different depth (layers are 6 μm apart) from the most anterior to the most posterior slice. Colors were assigned randomly to different components. Data can be found on CNCRS.org (http://dx.doi.org/10.6080/K01J97ZN). CNCRS, Collaborative Research in Computational Neuroscience; ICA, independent component analysis; NA, numerical aperture; PCA, principal component analysis. (TIF) [file pbio.2006732.s018.tif]

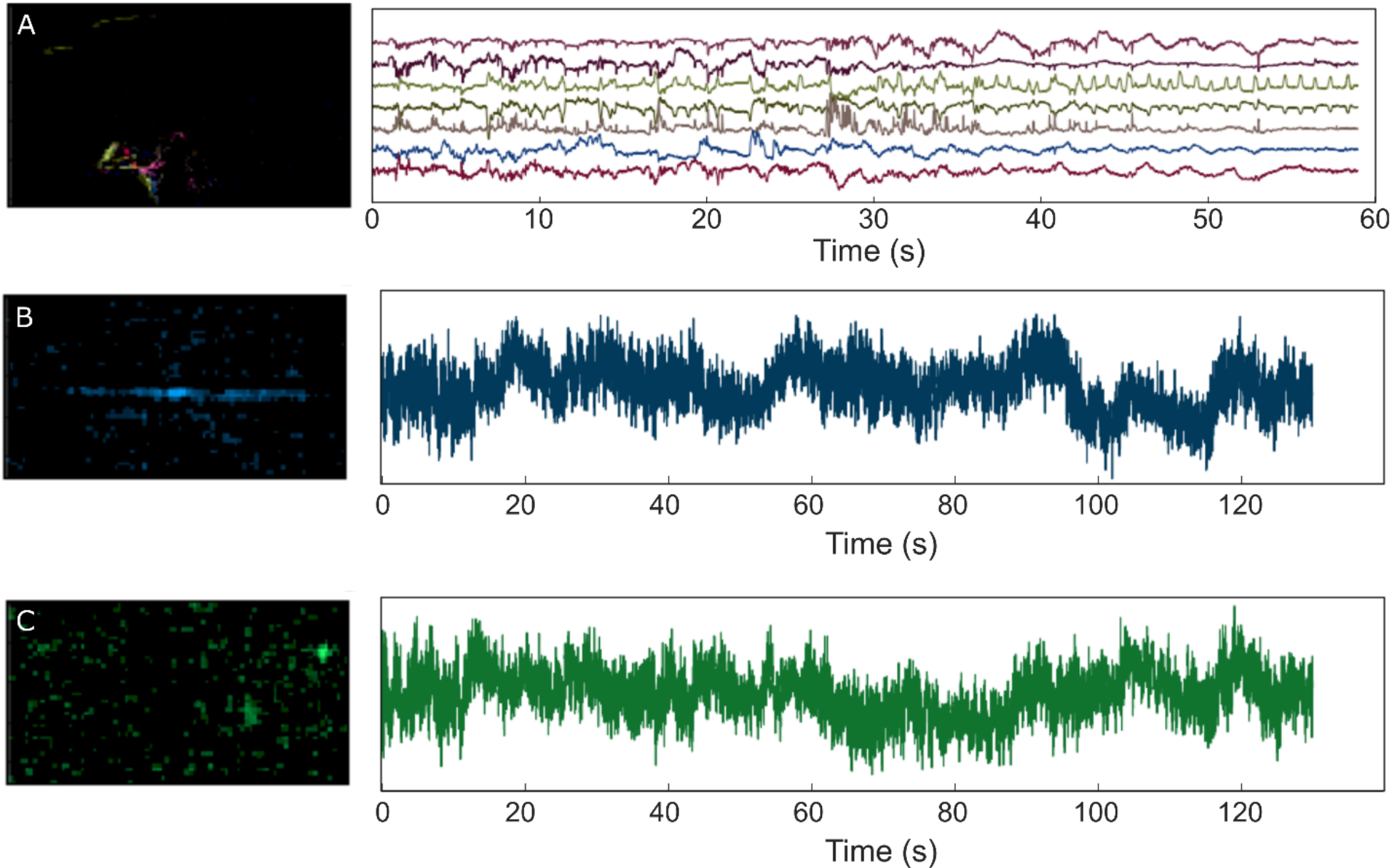

Supplement: S12 Fig — A) Movement artifacts: those components are present on the edge of the brain, and the time series have large transient uncharacteristic of activity probes. B) Horizontal lines resulting from aliasing after reconstruction. C) Background noise. GFP, green fluorescent protein. (TIF) [file pbio.2006732.s019.tif]

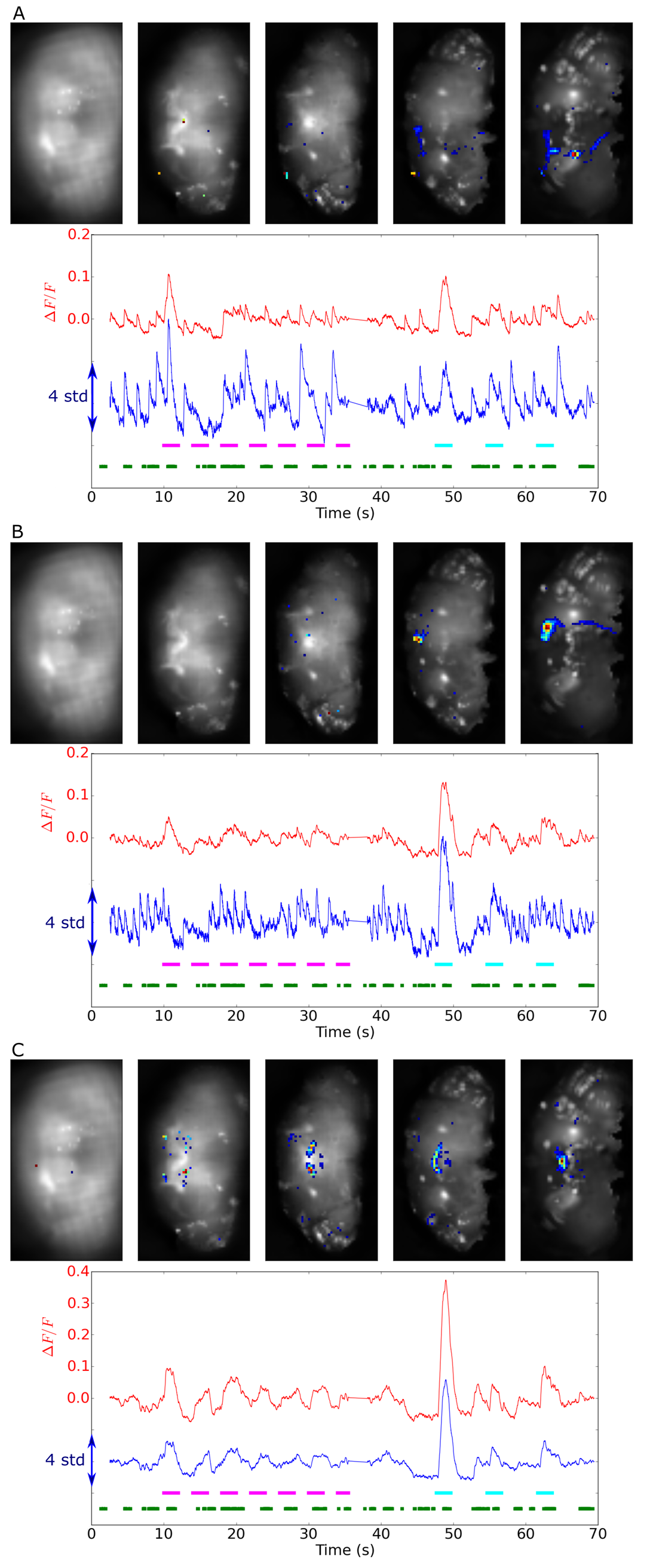

Supplement: S13 Fig — Red curve is the average of the fluorescence in the thresholded map, blue curve is the time series resulting from PCA/ICA, green dashes corresponds to the fly walking, cyan dashes to the presentation of odor puffs, and magenta dashes to the presentation of flashes of light. For components’ map with a thin track (A and B), possibly representing single neurons, PCA/ICA unmixes action potential-like signals from the background. For maps representing neuropil regions (such as a layer in the fan-Shaped body in C), ROI time series and PCA/ICA time series are very similar. Data can be found on CNCRS.org (http://dx.doi.org/10.6080/K01J97ZN). CNCRS, Collaborative Research in Computational Neuroscience; ICA, independent component analysis; PCA, principal component analysis; ROI, region of interest. (TIF) [file pbio.2006732.s020.tif]

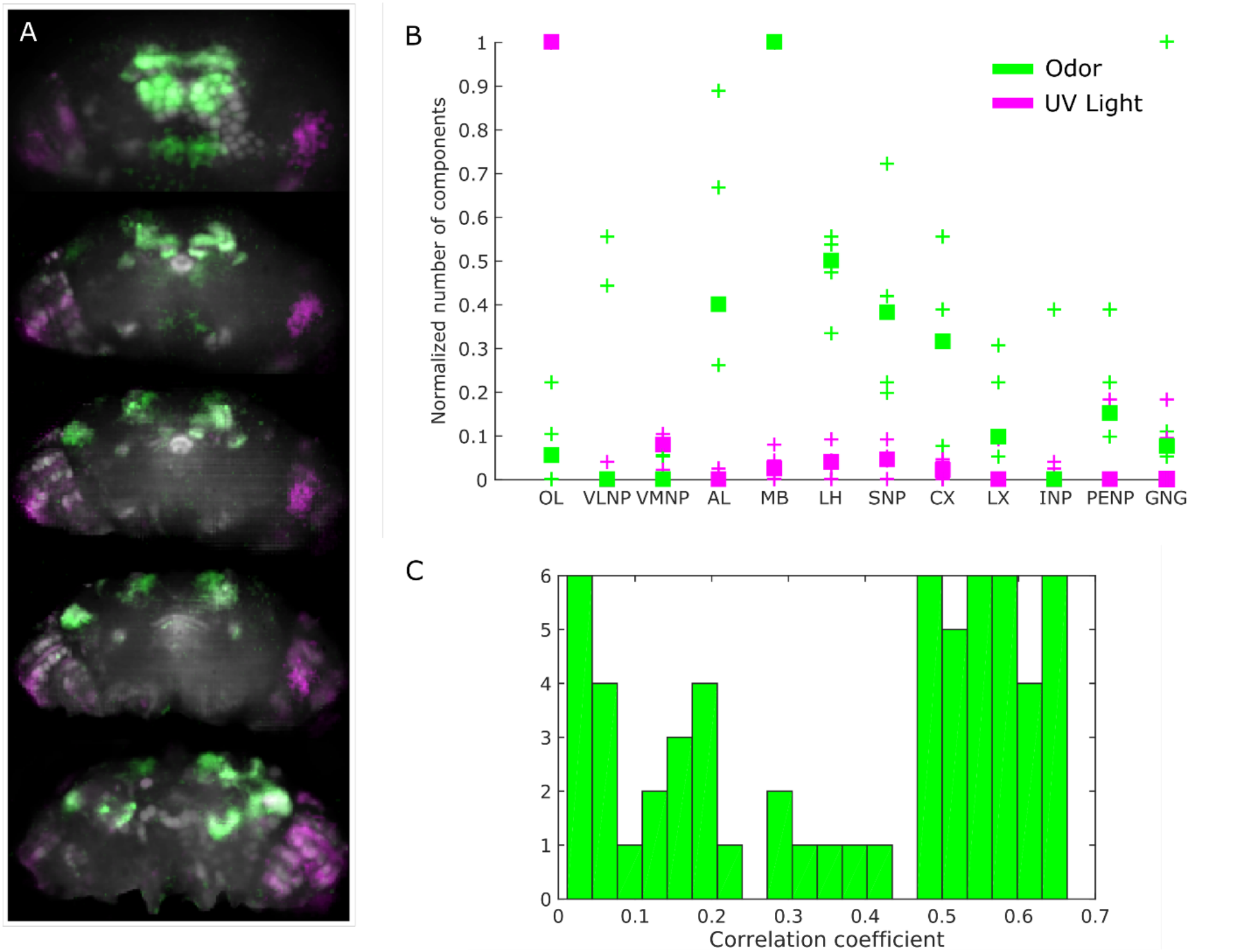

Supplement: S14 Fig — A) Maps’ z-stack of components correlated with the onset of UV light (magenta) or Odor (green). B) Number of components per brain region (normalized by the number for the region with the most components) that were correlated with the onset of UV light or the onset of odor (with correlation coefficient > 0.1) for flies expressing GCaMP6 pan-neuronally. Different points correspond to different flies (N = 5), and squares show the medians. C) Histogram of correlation coefficients (square root of R2) with odor onset convolved with a GCaMP6 kernel, for components present in all the three regions: antennal lobe, mushroom body calyx, and lateral horn, thus likely representing the activity of antennal lobe projection neurons. The underlying numerical values can be found in S1 Data. Data can also be found on CNCRS.org (http://dx.doi.org/10.6080/K01J97ZN). AL, antennal lobe; CNCRS, Collaborative Research in Computational Neuroscience; CX, central complex; GCaMP6F; GNG, gnathal ganglia; INP, inferior neuropils; LH, lateral horn; LX, lateral complex; MB, mushroom body; OL, optic lobe; PENP, periesophageal neuropils; SNP, superior neuropils; VMNP, ventromedial neuropils; VLNP, ventrolateral neuropils. (TIF) [file pbio.2006732.s021.tif]

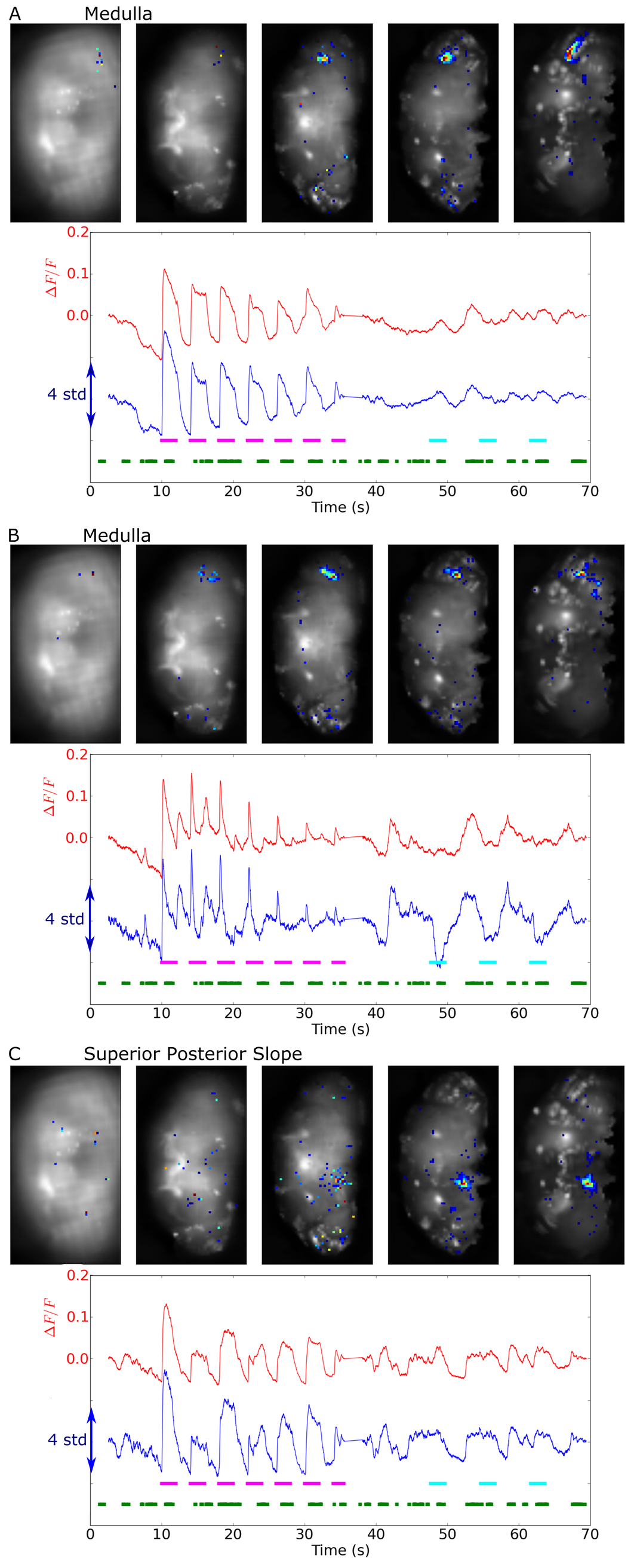

Supplement: S15 Fig — Red curve is the average of the fluorescence in the thresholded map, blue curve is the time series resulting from PCA/ICA, green dashes corresponds to the fly walking, cyan dashes to the presentation of odor puffs, and magenta dashes to the presentation of flashes of light. In C, note how the background transient corresponding to the first odor presentation is removed by PCA/ICA. ICA, independent component analysis; PCA, principal component analysis. (TIF) [file pbio.2006732.s022.tif]

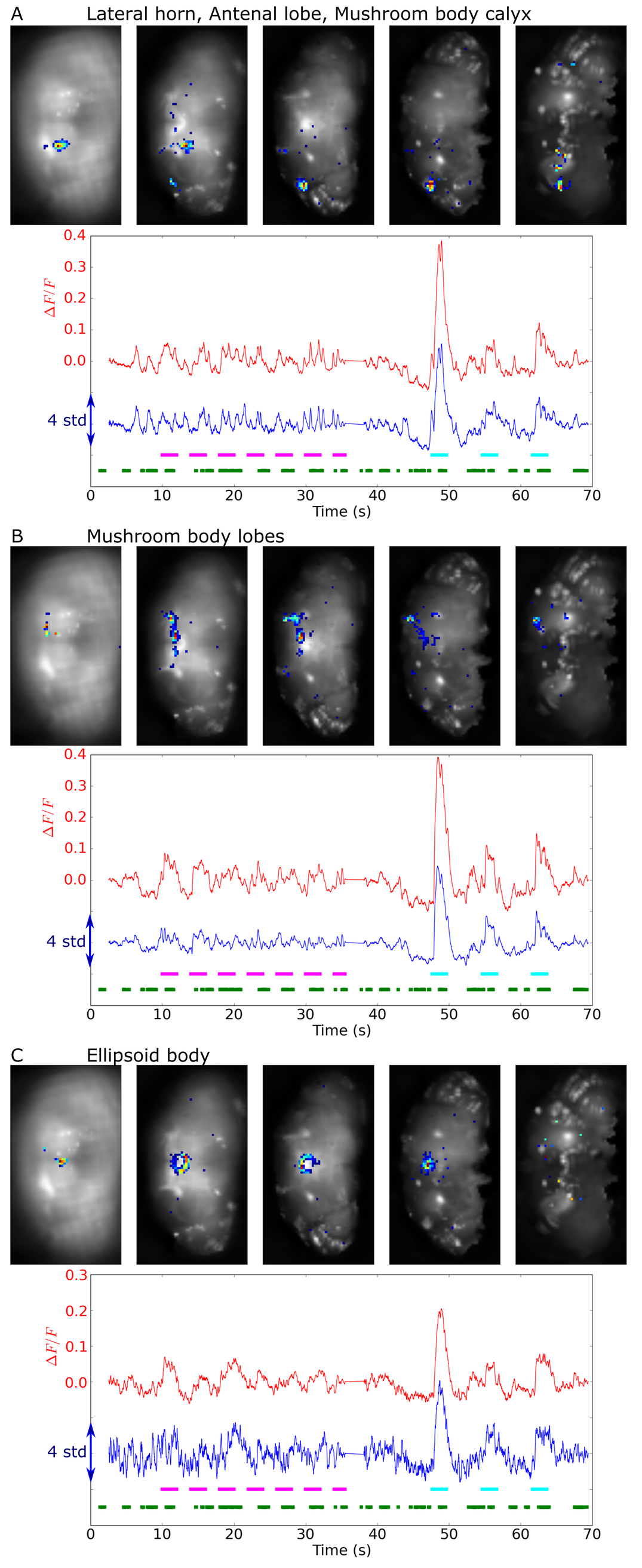

Supplement: S16 Fig — Red curve is the average of the fluorescence in the thresholded map (ROI), blue curve is the time series resulting from PCA/ICA, green dashes corresponds to the fly walking, magenta dashes to the presentation of flashes of light, and cyan dashes to the presentation of odor puffs. Note how background transients associated with the response to light (mainly above the first magenta dash) present in the ROI average are excluded from the components time series. Data can be found on CNCRS.org (http://dx.doi.org/10.6080/K01J97ZN). CNCRS, Collaborative Research in Computational Neuroscience; ICA, independent component analysis; PCA, principal component analysis; ROI, region of interest. (TIF) [file pbio.2006732.s023.tif]

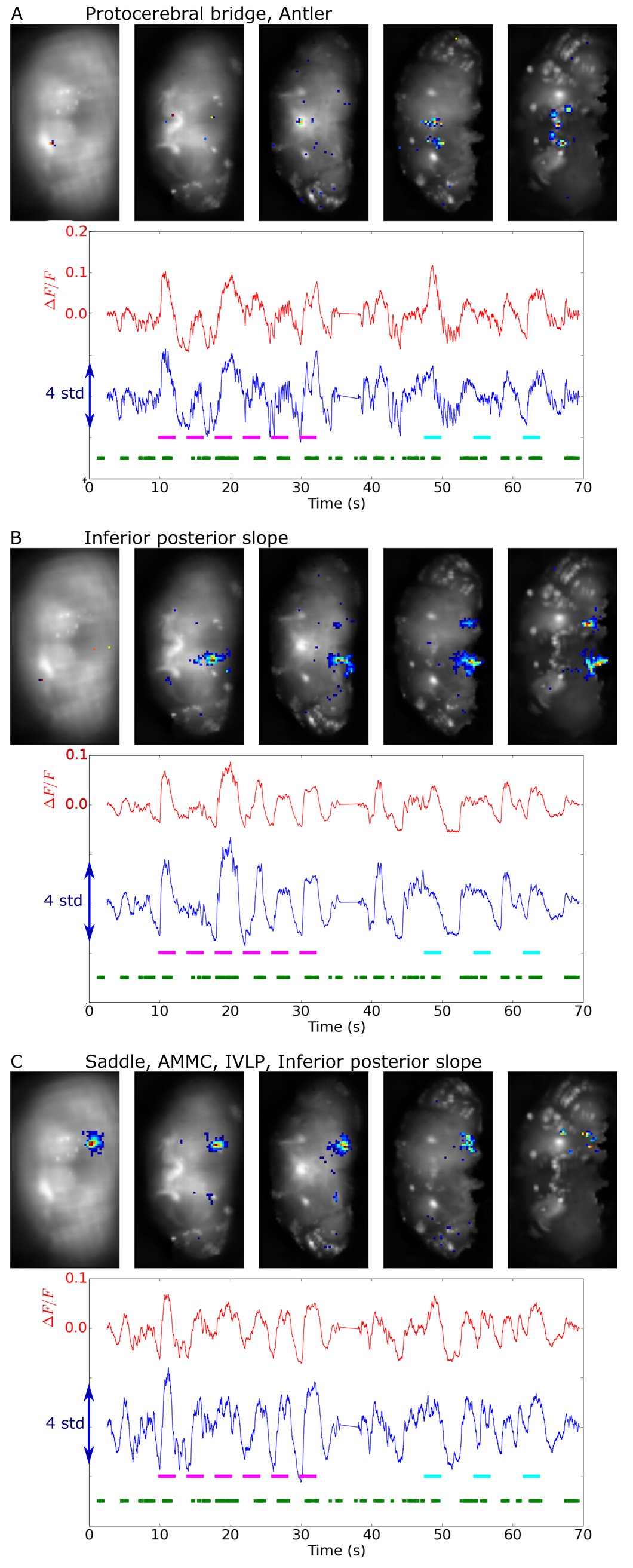

Supplement: S17 Fig — Red curve is the average of the fluorescence in the thresholded map (ROI), blue curve is the time series resulting from PCA/ICA, green dashes corresponds to the fly walking, magenta dashes to the presentation of flashes of light, and cyan dashes to the presentation of odor puffs. Note how the background transients associated with the response to odor in the ROI average are excluded from components time series. Data can be found on CNCRS.org (http://dx.doi.org/10.6080/K01J97ZN). CNCRS, Collaborative Research in Computational Neuroscience; ICA, independent component analysis; PCA, principal component analysis; ROI, region of interest. (TIF) [file pbio.2006732.s024.tif]

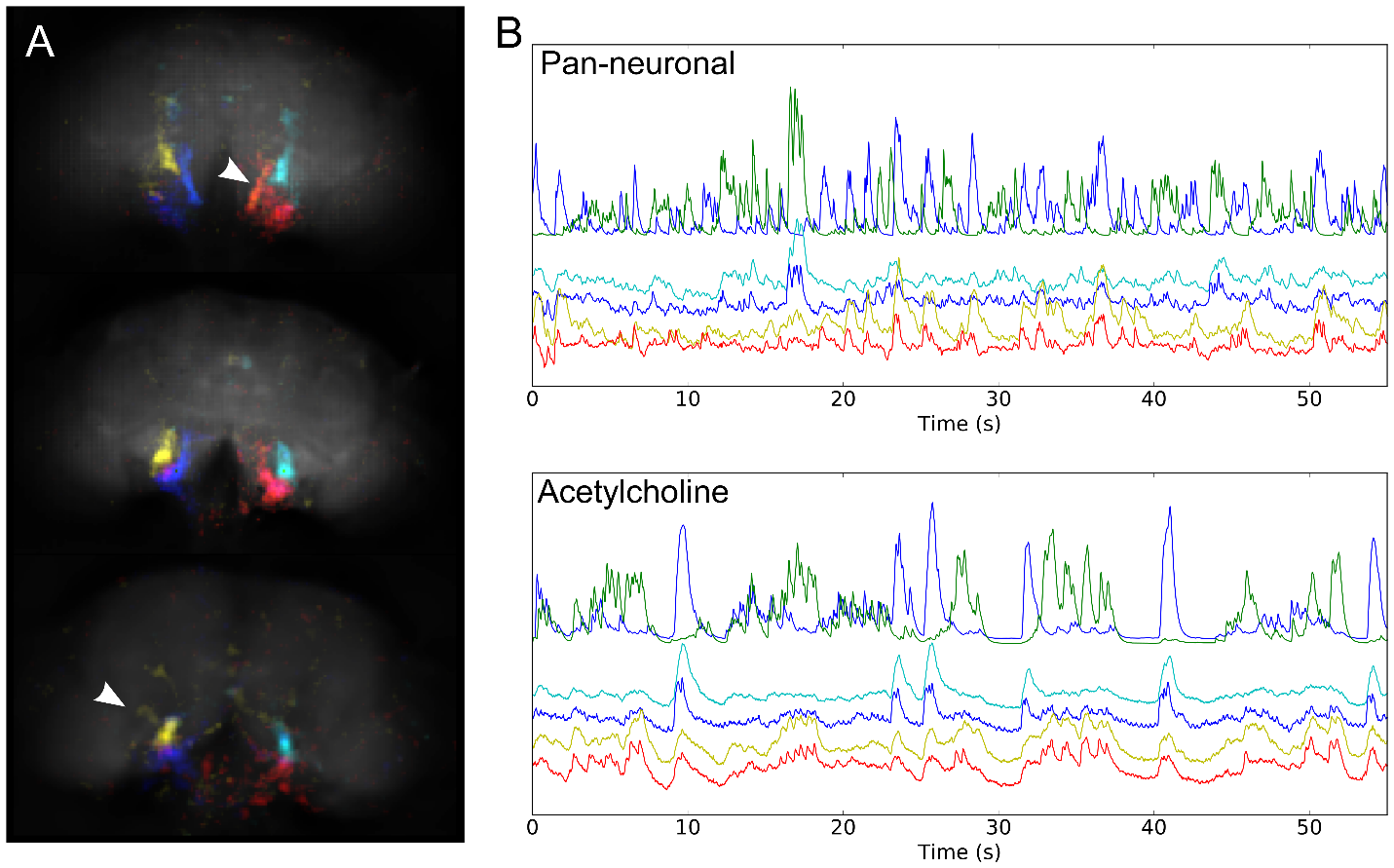

Supplement: S18 Fig — A) Anterior, medial and posterior slices of the components most correlated to turning left (cyan and blue) and right (red and yellow). The arrows point to characteristic shapes (inverted V or fine claw-like dorsal neurites). B) Example time series (variance normalized) for the four different components along with turning behavior. Top panel shows data from a pan-neuronal line (nsyb-Gal4, UAS-GCaMP6F), and bottom panel, from the same fly as A). In each panel, the top two traces (green and blue) are proportional to angular speed of the ball towards left or right, convolved with a GCaMP6 kernel. The four bottom traces show activity components (same color code as the maps). Note that the cyan (or yellow) trace is slightly delayed compared to the blue trace (or red trace respectively). Cha-Gal4, choline acetyltransferase-Gal4; nsyb-Gal4, nSynaptobrevin-Gal4. (TIF) [file pbio.2006732.s025.tif]

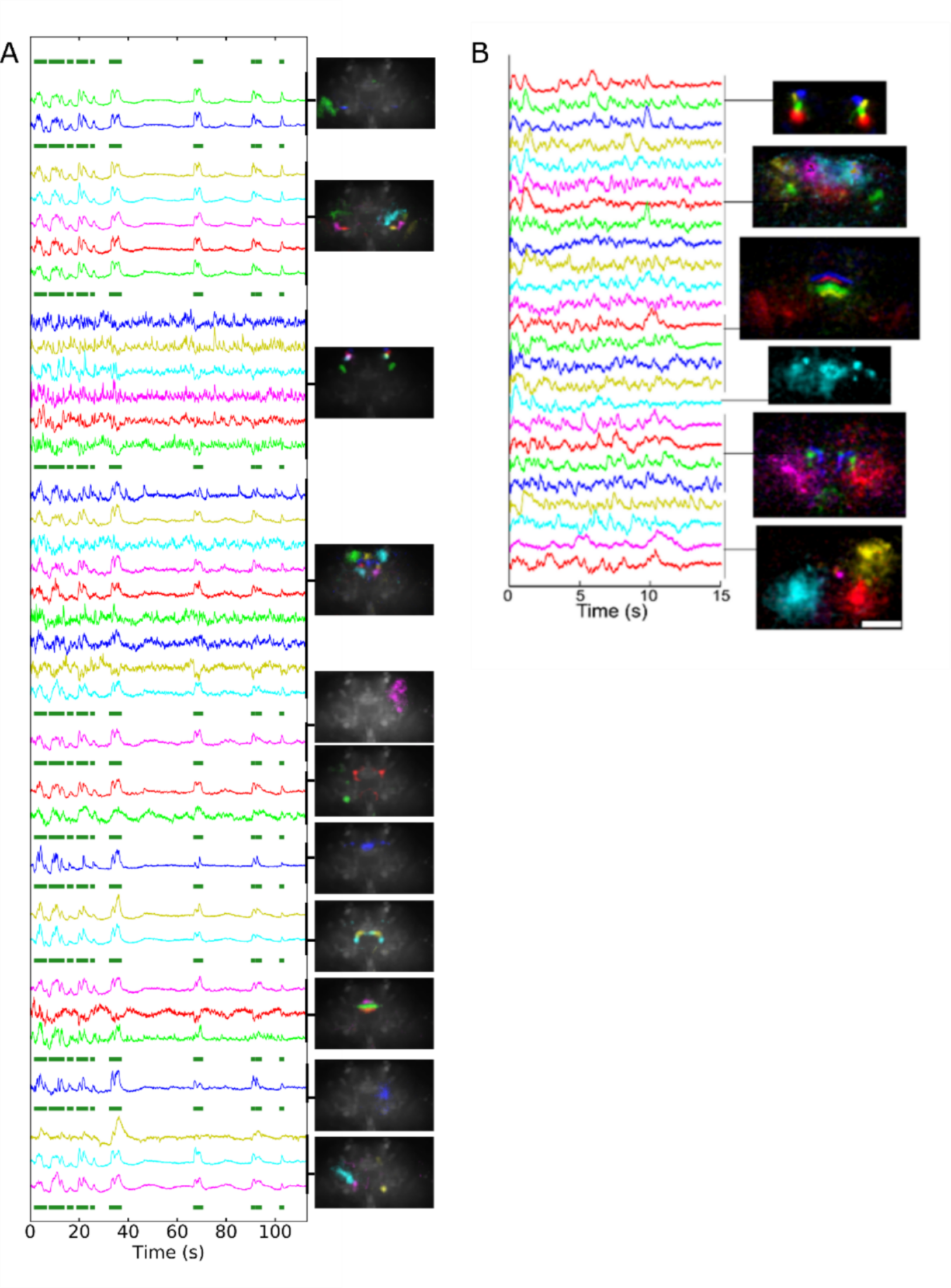

Supplement: S19 Fig — Left: components’ time series (variance normalized), right: components’ map. A and B are different flies. In A, the green dashes correspond to the fly walking. TH-Gal4, tyrosine hydroxylase-Gal4. (TIF) [file pbio.2006732.s026.tif]

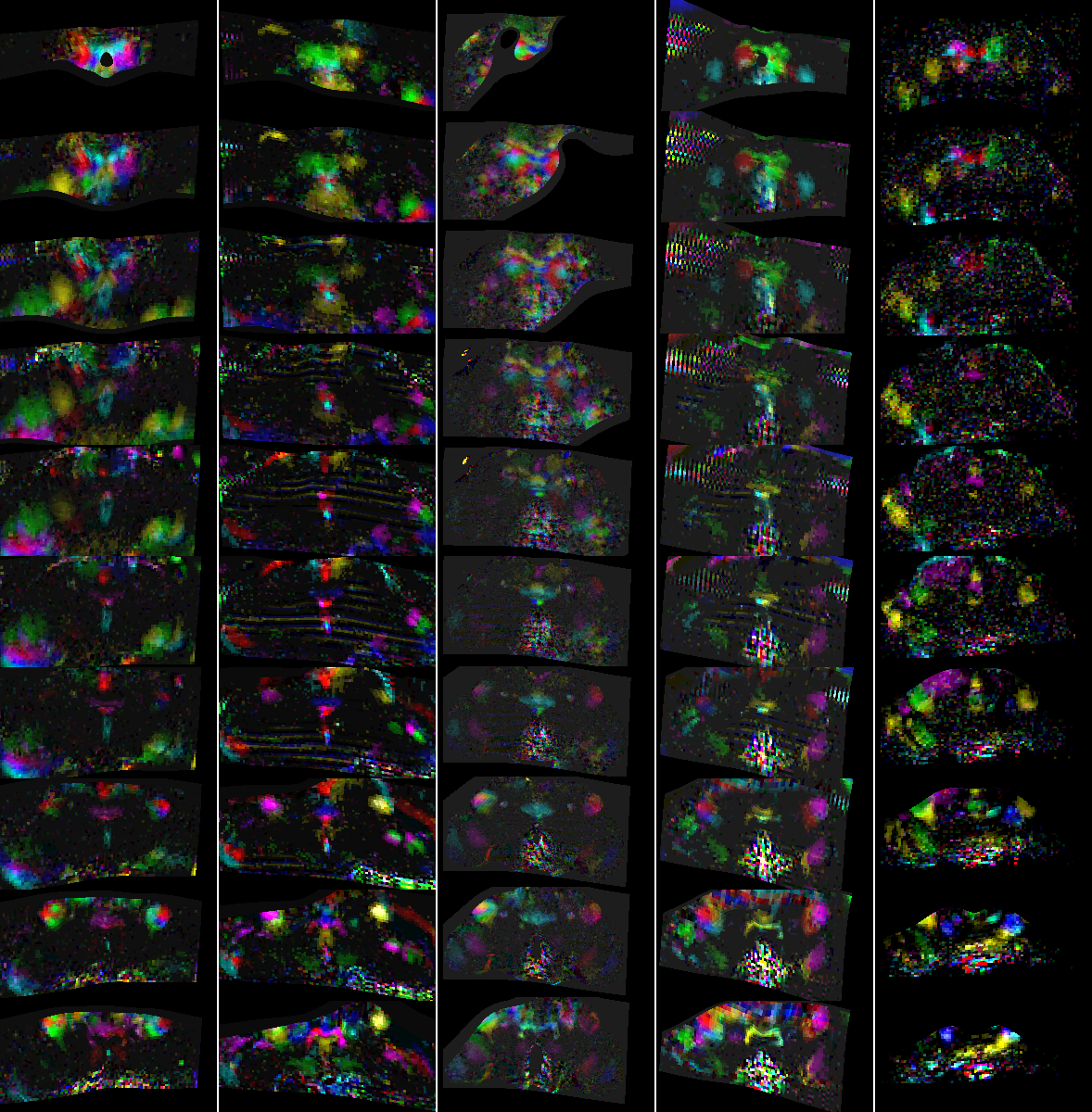

Supplement: S20 Fig — The maps were aligned to an anatomical template. Each column presents a z-stack of the 3D map for one fly. Each column corresponds to a different fly, and rows correspond to different depth (6 μm apart), from the most anterior slice at the top to the most posterior at the bottom. Colors were assigned randomly to different components. Data can be found on CNCRS.org (http://dx.doi.org/10.6080/K01J97ZN). CNCRS, Collaborative Research in Computational Neuroscience; NA, numerical aperture; ICA, independent component analysis; PCA, principal component analysis. (TIF) [file pbio.2006732.s027.tif]

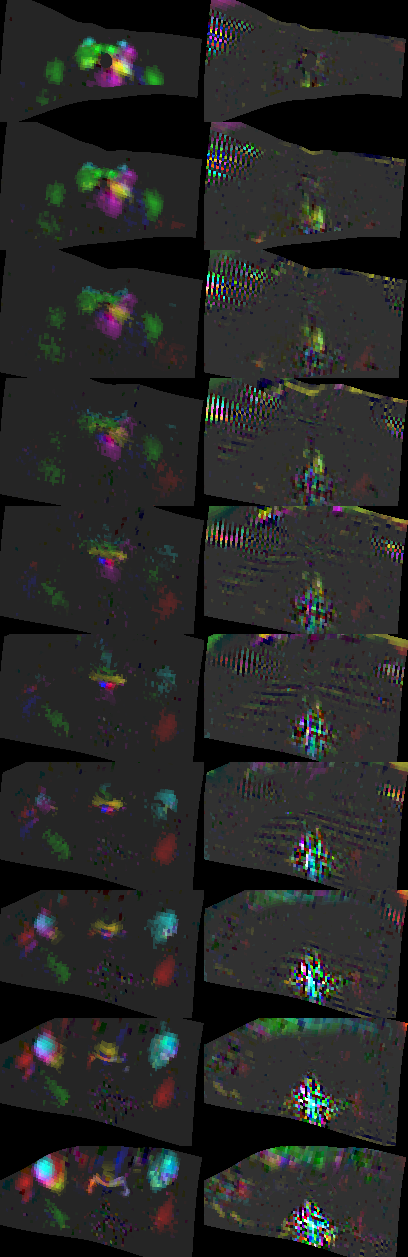

Supplement: S21 Fig — Left is a z-stack of activity-related components and right are artifactual components. (TIF) [file pbio.2006732.s028.tif]

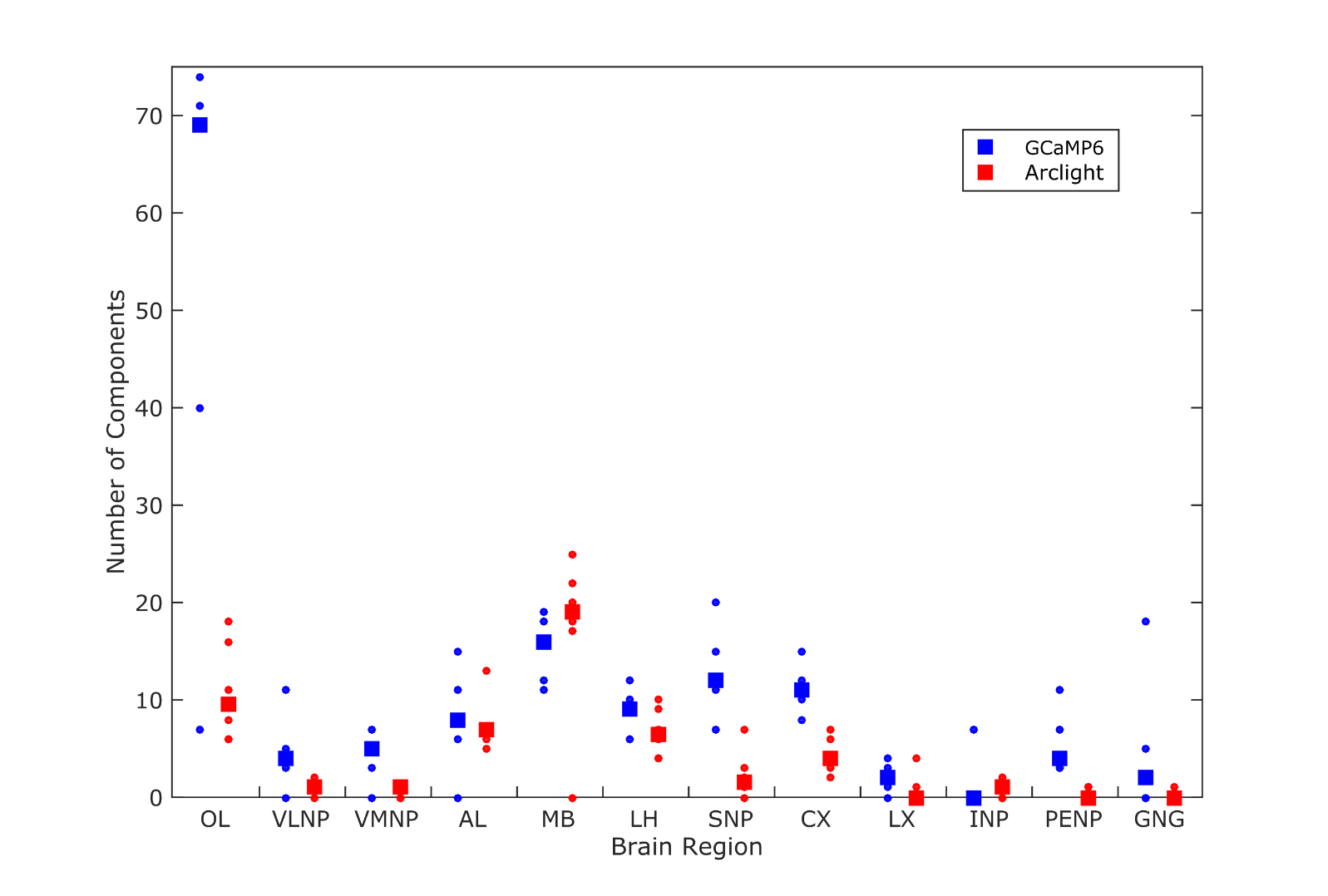

Supplement: S22 Fig — In all experiments, puffs of odor and flashes of UV light were presented to the flies (N = 6 for ArcLight and N = 5 for GCaMP6). Dots are for single flies and the squares represent the medians. The underlying numerical values can be found in S1 Data. AL, antennal lobe; CX, central complex; GNG, gnathal ganglia; INP, inferior neuropils; LH, lateral horn; LX, lateral complex; MB, mushroom body; OL, optic lobe; PENP, periesophageal neuropils; SNP, superior neuropils; VMNP, ventromedial neuropils; VLNP, ventrolateral neuropils. (TIF) [file pbio.2006732.s029.tif]

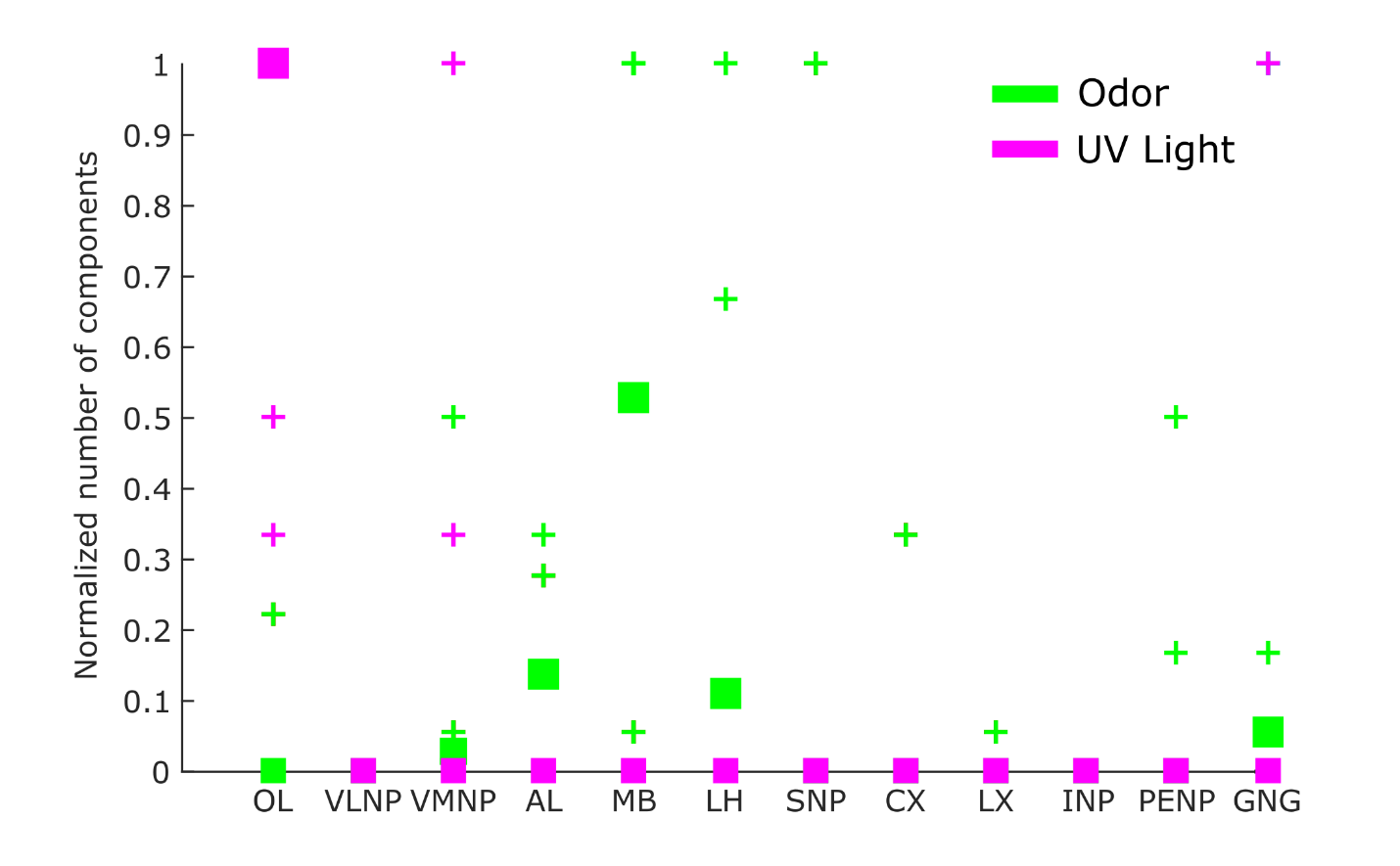

Supplement: S23 Fig — Different points correspond to different flies (N = 6), and squares indicate the medians. The underlying numerical values can be found in S1 Data. AL, antennal lobe; CX, central complex; GNG, gnathal ganglia; INP, inferior neuropils; LH, lateral horn; LX, lateral complex; MB, mushroom body; OL, optic lobe; PENP, periesophageal neuropils; SNP, superior neuropils; VMNP, ventromedial neuropils; VLNP, ventrolateral neuropils. (TIF) [file pbio.2006732.s030.tif]

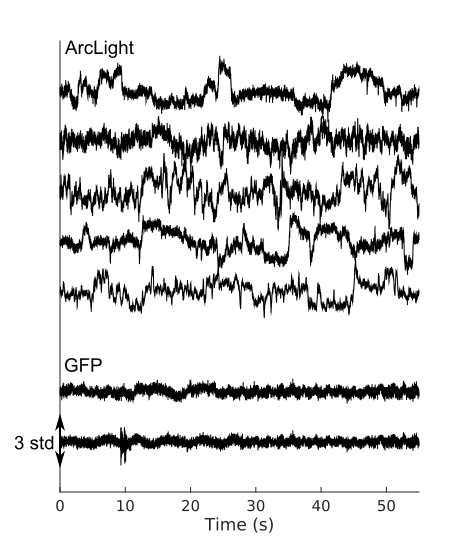

Supplement: S24 Fig — Because no such components were automatically extracted with pan-neuronally expressed GFP, we created masks of one nodulus and the opposite side of the protocerebral bridge using an anatomical template for the GFP control. GFP, green fluorescent protein. (TIF) [file pbio.2006732.s031.tif]

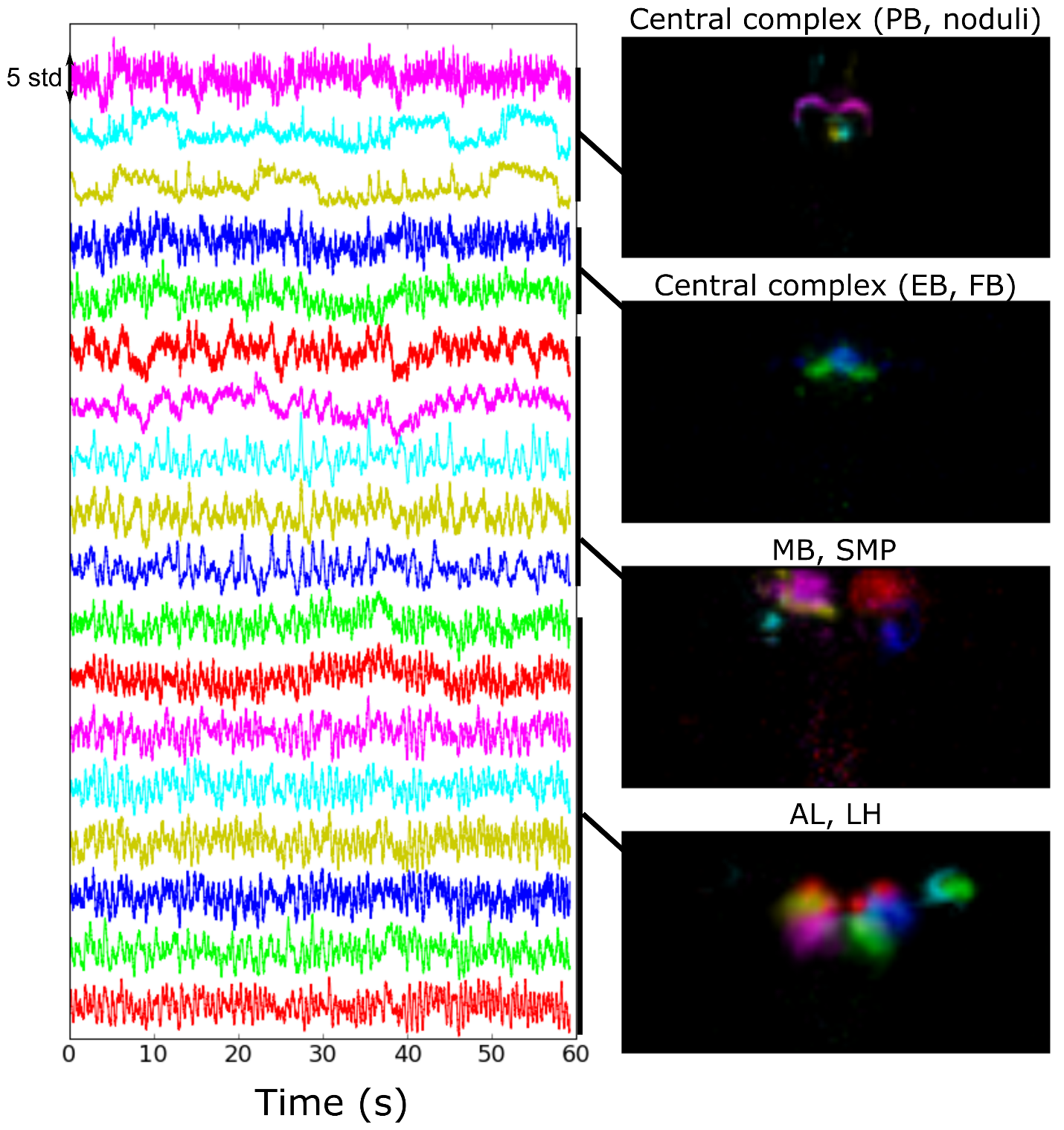

Supplement: S25 Fig — Left are the component’s time series (variance normalized) and right are the corresponding maps. Note the slow oscillations in the antennal lobe and lateral horn (peak frequency of the average power spectrum smoothed over 50 points is 1.6 Hz). The cumulative power spectrum reaches 80% at 3.6 Hz for those components but only at 8.6 Hz for components with faster ongoing activity such as the first and fourth components, in the protocerebral bridge, and the ellipsoid body. Data can be found on CNCRS.org (http://dx.doi.org/10.6080/K01J97ZN). Cha-Gal4, choline acetyltransferase-Gal4; CNCRS, Collaborative Research in Computational Neuroscience. (TIF) [file pbio.2006732.s032.tif]

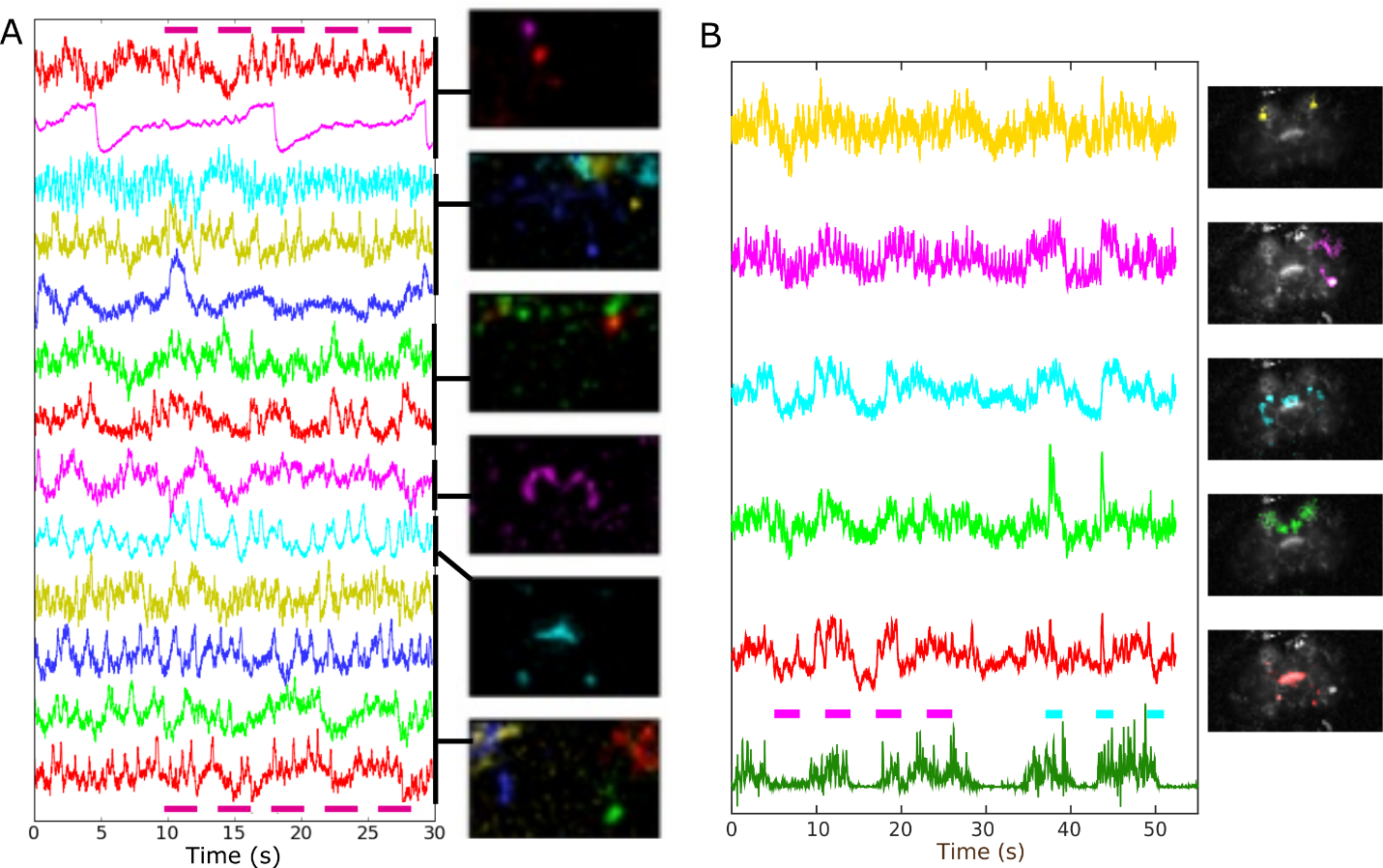

Supplement: S26 Fig — Left are the component’s variance normalized time series (magenta dashes correspond to the presentation of a flash of light, cyan dashes to puffs of odor, and forest green traces to walking speed) and right are the corresponding z-projected maps. In A), for example, the first two time series correspond to the first image; the next three time series to the second image. In B), each time series corresponds to one map showed on the right. DDC-Gal4, Dopa decarboxylase-Gal4; TH-Gal4, tyrosine hydroxylase-Gal4. (TIF) [file pbio.2006732.s033.tif]

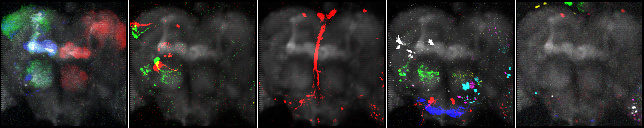

Supplement: S27 Fig — Different panels present a maximum z projection for different types of components as described in the text, and in each panel different colors correspond to different individual components. ICA, independent component analysis; PCA, principal component analysis. (TIF) [file pbio.2006732.s034.tif]

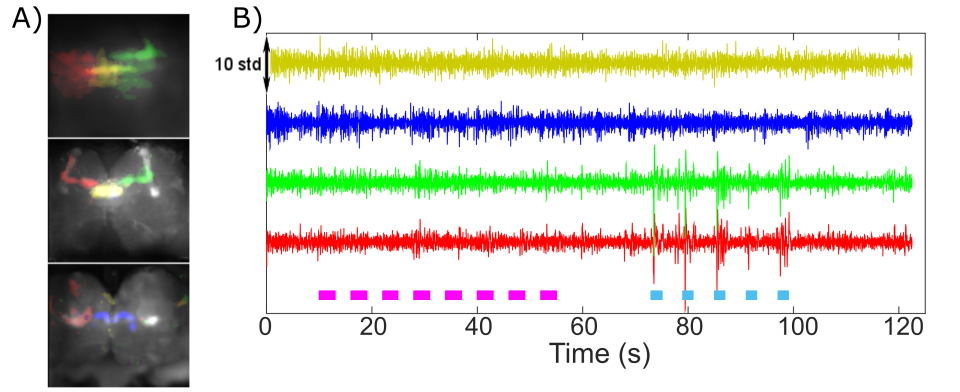

Supplement: S28 Fig — Magenta dashes correspond to flashes of UV light and cyan dashes to puffs of odors. Cha-Gal4, choline acetyltransferase-Gal4. (TIF) [file pbio.2006732.s035.tif]

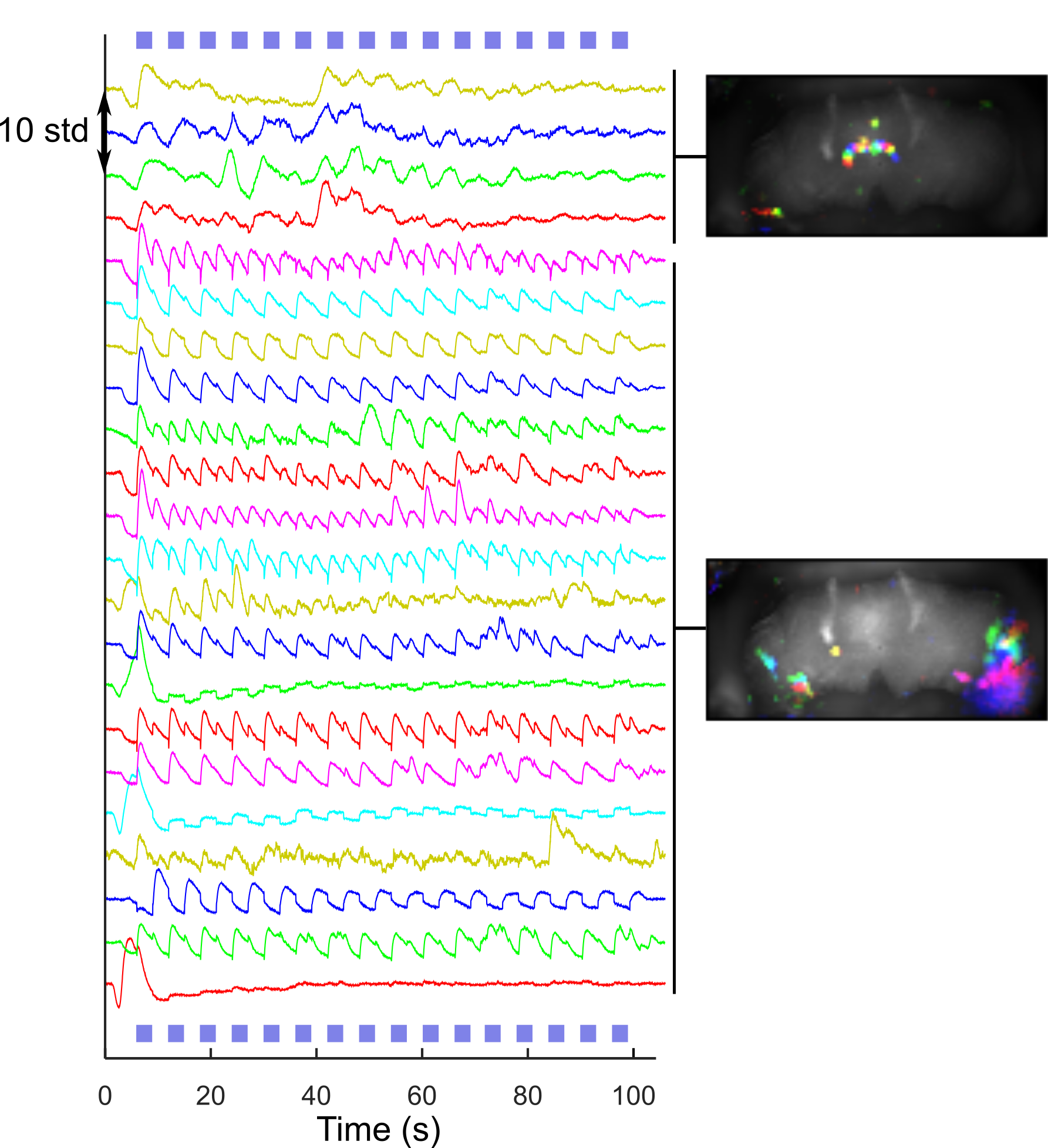

Supplement: S29 Fig — Only the components responding to the stimulus are included. Left are the component’s variance normalized time series and right are the corresponding z-projected maps. Blue dashes correspond to flashes of blue light. nsyb-Gal4, nSynaptobrevin-Gal4. (TIF) [file pbio.2006732.s036.tif]
